# Aptamer-Modified Cu^2+^-Functionalized C-Dots: Versatile Means to Improve Nanozyme Activities-“Aptananozymes”

**DOI:** 10.1021/jacs.1c03939

**Published:** 2021-07-21

**Authors:** Yu Ouyang, Yonatan Biniuri, Michael Fadeev, Pu Zhang, Raanan Carmieli, Margarita Vázquez-González, Itamar Willner

**Affiliations:** † The Institute of Chemistry, The Hebrew University of Jerusalem, Jerusalem 91904, Israel; ‡ Department of Chemical Research Support, Weizmann Institute of Science, Rehovot 76100, Israel

## Abstract

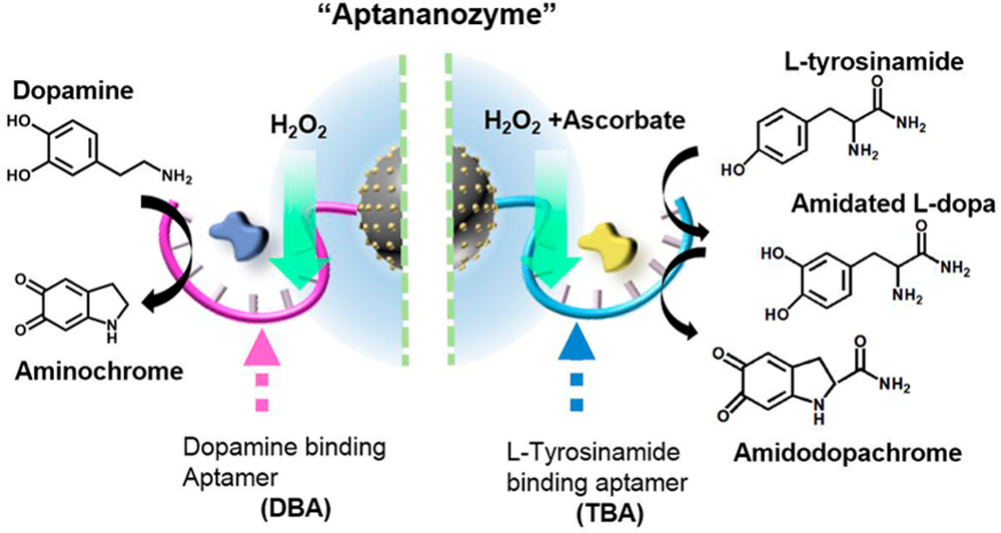

The covalent linkage of aptamer binding sites to nanoparticle nanozymes is introduced as a versatile method to improve the catalytic activity of nanozymes by concentrating the reaction substrates at the catalytic nanozyme core, thereby emulating the binding and catalytic active-site functions of native enzymes. The concept is exemplified with the synthesis of Cu^2+^ ion-functionalized carbon dots (C-dots), modified with the dopamine binding aptamer (DBA) or the tyrosinamide binding aptamer (TBA), for the catalyzed oxidation of dopamine to aminochrome by H_2_O_2_ or the oxygenation of l-tyrosinamide to the catechol product, which is subsequently oxidized to amidodopachrome, in the presence of H_2_O_2_/ascorbate mixture. Sets of structurally functionalized DBA-modified Cu^2+^ ion-functionalized C-dots or sets of structurally functionalized TBA-modified Cu^2+^ ion-functionalized C-dots are introduced as nanozymes of superior catalytic activities (aptananozymes) toward the oxidation of dopamine or the oxygenation of l-tyrosinamide, respectively. The aptananozymes reveal enhanced catalytic activities as compared to the separated catalyst and respective aptamer constituents. The catalytic functions of the aptananozymes are controlled by the structure of the aptamer units linked to the Cu^2+^ ion-functionalized C-dots. In addition, the aptananozyme shows chiroselective catalytic functions demonstrated by the chiroselective-catalyzed oxidation of l/d-DOPA to l/d-dopachrome. Binding studies of the substrates to the different aptananozymes and mechanistic studies associated with the catalytic transformations are discussed.

## Introduction

Substantial research efforts are directed to the development of inorganic, organic, or metal–organic framework nanoparticles that mimic the functions of native enzymes, “nanozymes”.^1^ Inorganic nanoparticles, such as Fe_3_O_4_,^2^ V_2_O_5_,^3^ CeO_2_,^4^ MoO_3_,^5^ Au,^6^ Ag,^7^ Pd,^8^ carbon-based materials, for example, carbon-dots (C-dots)^9^ or graphene quantum dots,^10^ Prussian Blue nanoparticles,^11^ organic nanoparticles, such as melanin nanoparticles,^12^ and metal–organic framework nanoparticles (NMOFs), such as Zr-based NMOFs^13^ or MOF-818,^14^ were reported as nanozymes. Different chemical transformations emulating native enzymes were demonstrated by synthetic nanozymes including oxidase,^15^ peroxidase,^16^ laccase,^17^ catalase,^18^ superoxide dismutase,^19^ and hydrolase activities.^20^ Diverse applications of nanozymes were reported including their use as sensors,^21^ imaging agents,^22^ biomedical applications,^23^ such as cancer therapies,^24^ and the treatment of other diseases, for example, Alzheimer’s^25^ or Parkinson’s diseases.^26^ In addition, nanozymes were applied as antibacterial agents^27^ and materials for environmental degradation of pollutants.^28^

In contrast to native enzymes exhibiting active sites composed of recognition binding sites and catalytic units that cooperatively lead to superior catalytic activities, nanozymes lack the substrate binding feature and concentration of the substrate at the catalytic interface (lack of high molarity at the catalytic sites). Thus, the development of a versatile means to concentrate the substrates at the nanozymes catalytic surface could be a major advance in the field of nanozymes. Several approaches to reach these goals were reported including the functionalization of nanozymes with β-cyclodextrin receptors^9^ or the coating of the nanocatalysts with molecularly imprinted matrices.^29^

Aptamers are sequence-specific nucleic acids that are selected by the systematic evolution of ligands by exponential enrichment (SELEX) procedure.^30^ Aptamers revealing high affinity and selective binding properties toward low-molecular-weight ligands and macromolecules were developed.^31^ These selective binding properties were extensively used for analytical applications, for example, the development of sensors,^32^ separation matrices,^33^ and for biomedical applications,^34^ such as stimuli-responsive drug carriers,^35^ functional protein inhibitors,^36^ targeting of cancer cells,^37^ and imaging^38^ of cells. Also, the binding and dissociation of aptamer/ligand complexes were used to switch DNA nanostructures and to trigger DNA machines.^39^ Thus, the conjugation of ligand-binding aptamers to nanozymes could provide a versatile means to mimic native enzymes by concentrating ligand-substrate complexes at the catalytic sites of nanozymes.

In the past few years, we developed the concept of nucleoapzymes, where aptamers were conjugated to catalytic nucleic acids or homogeneous catalysts, as a versatile approach to yield hybrid homogeneous catalytic structures mimicking a native enzyme.^40−43^ The versatility and diversity of this approach were reflected by the possibility to link the catalytic units to the 5′ or 3′ end of the aptamers and to link the catalyst to the aptamer through spacer bridges or to position the catalyst in middle positions of the aptamers. These catalyst-aptamer structures have established a small library of sets of nucleoapzymes revealing variable catalytic activities originating from different binding affinities of the substrates to the modified aptamers or from steric constraints dictating the spatial positioning of the catalytic sites in respect to the binding sites. Molecular dynamic simulations were applied to elucidate the structure–function relationships within the catalyst-aptamer conjugates to account for the variable catalytic activities of the nucleoapzyme structures. Different nucleoapzymes catalyzing the oxidation of dopamine or *N*-hydroxy-l-arginine by H_2_O_2_ to yield aminochrome or citruline, respectively,^40^ the hydrolysis of ATP to ADP by mimicking ATPase,^42^ and the oxygenation of aryl C–H bonds of l-tyrosinamide to yield its catechol derivatives^43^ have been demonstrated. In principle, this concept can be applied to develop libraries of aptamer-nanozyme hybrids of variable catalytic activities by the conjugation of the 3′ or 5′ end of the aptamer to the nanozyme, the introduction of spacer units that modulate the flexibility of the aptamers in respect to the heterogeneous nanozyme, and by further engineering the DNA bridging modules linking the aptamers to the nanozymes, for example, by introducing stimuli-responsive bridging units. Although substantial advances in developing homogeneous catalytic conjugates consisting of catalyst-aptamer conjugates were demonstrated, the analogous heterogeneous catalyst-aptamer conjugates as functional catalytic units are unprecedented. Thus, realizing the limitations of catalytic nanoparticles (nanozymes) that lack substrate concentration sites, the conjugation of aptamers to the nanozymes could yield a versatile approach to synthesize heterogeneous catalytic modules, thereby bringing homogeneous catalyst-aptamer structures with heterogeneous aptamer hybrid as a unified approach for DNA-based catalytic structures. Realizing the topic of nanozymes is a rapidly developing area in catalysis, we believe that the integration of aptamers into heterogeneous catalysts as selective substrate binding and concentration means yields a new insight into heterogeneous nanozyme catalysis.

In the present study, we describe the conjugation of the dopamine binding aptamer (DBA) or the l-tyrosinamide binding aptamer (TBA) to Cu^2+^ ion-functionalized C-dots. We demonstrate that the anchoring mode of the aptamer and the spacers separating the aptamer from the catalyst affect the resulting catalytic activities of the aptamer-nanozyme conjugates (aptananozymes). We believe that the present study paves a versatile approach to develop nanozymes of enhanced catalytic performances.

## Resulsts and Discussion

**Figure 1:**
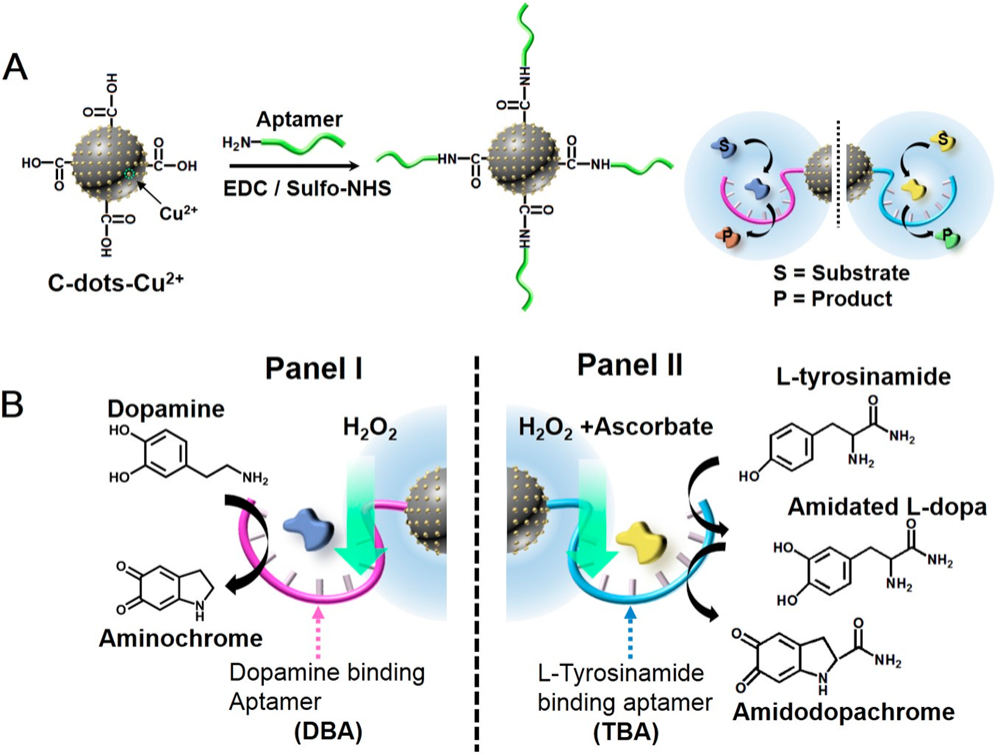
(A) Schematic synthesis of aptamer-modified Cu^2+^ ion-functionalized C-dots-aptananozyme. (B) Schematic chemical transformations driven by the synthetic aptananozymes. (Panel I) Catalyzing the oxidation of dopamine. (Panel II) Catalyzing oxygenation and oxidation of tyrosinamide.

Carbon dots, C-dots, were prepared by a microwave treatment of mixture of citric acid and urea, according to the reported procedure.^44^ The resulting C-dots (ca. 10 nm diameter, Figure S1) include carboxylic acid and amine functionalities on their surface. The C-dots were functionalized with Cu^2+^ ions through coordination interactions. X-ray photoelectron spectroscopy (XPS) measurements confirmed the association of Cu^2+^ ions to the C-dots, and Fourier-transform infrared spectroscopy (FTIR) indicated the formation of metal–ligand (Cu–N, 1045 cm^–1^)^45^ bonds on the surface of the C-dots particles, Figures S2 and S3. The binding of Cu^2+^ ions to C-dots support yields a stable complex, *K*_d_ = 10.5 ± 1.2 nM. Figure S4 shows the experimental curves corresponding to the isothermal titration calorimetry (ITC) for evaluating the dissociation constants. In addition, inductively coupled plasma mass spectrometry (ICP-MS) measurements analyzing possibly released Cu^2+^ ions to the bulk solution indicated only trace concentrations (15 ng mL^–1^) and even higher concentration of Cu^2+^ ions in the bulk solution that did not reveal any catalytic performance on the substrates described in the study, Figure S9C. The respective amino-functionalized DBA^46^ or TBA^43^ were covalently coupled to the free carboxylic acid functionalities associated with the Cu^2+^ ion-functionalized C-dots using 1-ethyl-3-(3-(dimethylamino)propyl) carbodiimide (EDC) and *N*-hydroxysulfosuccinimide (sulfo-NHS) as coupling reagents. The surface coverage of the different aptamers on the Cu^2+^ ion-functionalized C-dots was evaluated spectroscopically; see Figure S5 and the accompanying discussion. The loading degrees of all aptamers used in the study were very similar and corresponded to four aptamer strands per C-dot. ICP-MS analyses indicated that the loading of Cu^2+^ ions corresponded to 40 ± 3 μg per mg of C-dots, Table S1. The amino-aptamer-modified Cu^2+^ ion-functionalized C-dots were then applied to examine the catalyzed oxidation of dopamine to aminochrome by H_2_O_2_ in the presence of the DBA-modified Cu^2+^ ion-functionalized C-dots and the catalyzed oxygenation and oxidation of l-tyrosinamide to amidodopachrome by H_2_O_2_ and ascorbate (AA^–^), in the presence of the TBA-modified Cu^2+^ ion-functionalized C-dots, as schematically outlined in Figure 1.

**Figure 2:**
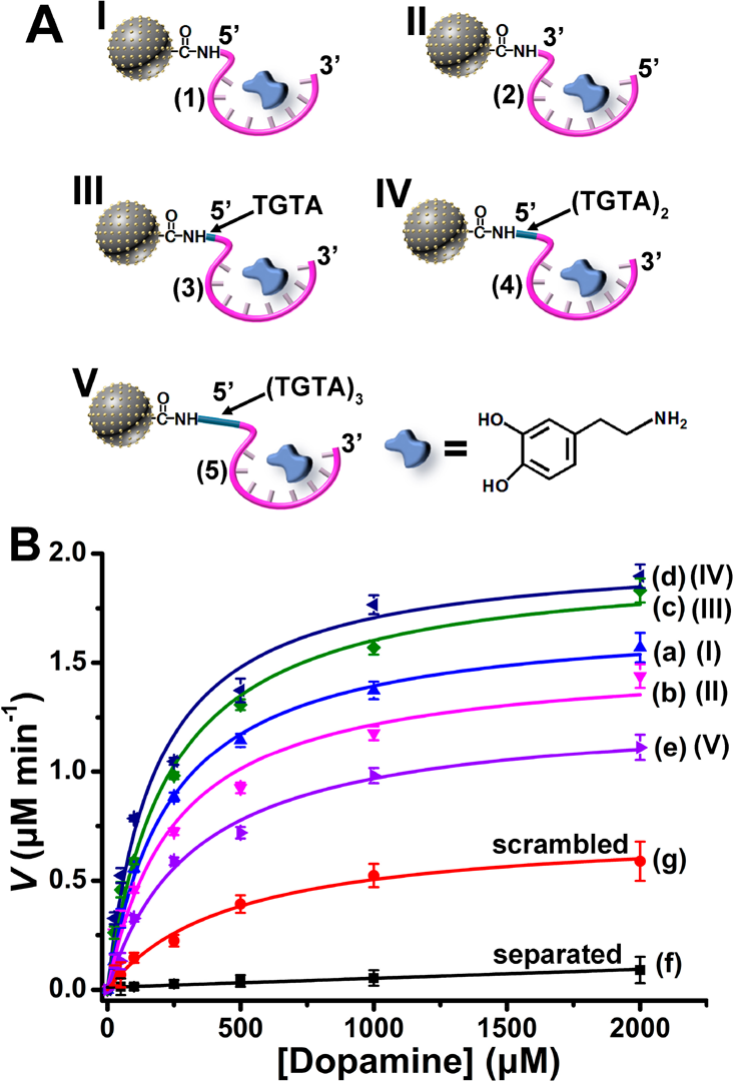
(A) The set of aptananozymes used for the catalyzed oxidation of dopamine to aminochrome (For the detailed sequences of the aptamers conjugated to the Cu^2+^ ion-functionalized C-dots see the Experimental Section). (B) Rates of oxidation of dopamine to aminochrome by H_2_O_2_ using variable concentrations of dopamine in the presence of the set of aptananozymes and the respective control systems. Error bars derived from *N* = 3 experiments.

A small library of DBA-modified Cu^2+^ ion-functionalized C-dots was prepared, Figure 2A, that included the 5′-end-amino-DBA (**1**) and 3′-end-amino-DBA (**2**) as aptamer-modified Cu^2+^ ion-functionalized C-dots acting as aptananozymes I and II. In addition, aptananozymes consisting of the 5′-end-amino-DBA linked to the Cu^2+^ ion-functionalized C-dots through spacer units were synthesized and included a (TGTA) spacer, aptananozyme III, (TGTA)_2_ spacer, aptananozyme IV, or (TGTA)_3_ spacer, aptananozyme V. The oxidation rates of dopamine to aminochrome by H_2_O_2_, in the presence of a constant weight of the respective DBA-modified Cu^2+^ ion-functionalized C-dots, were examined at variable concentrations of dopamine (in all of the experiments, H_2_O_2_, 5 mM, was used). The time-dependent absorbance changes corresponding to aminochrome formation were applied to follow the kinetics of dopamine oxidation by the respective aptananozymes (e.g., the time-dependent absorption spectra generated by aptananozyme (I) upon oxidation of dopamine to aminochrome, Figure S6, and the time-dependent absorbance changes at λ = 480 nm for the entire set of aptananozymes, Figure S7, panels i–vi). These kinetic data were used to derive the rates of oxidation of dopamine to aminochrome as a function of dopamine concentration, as outlined in Figure 2B, curves (a) to (e). For all aptananozymes, Michaelis–Menten-type saturation kinetic curves are observed. For comparison, the rates of oxidation of dopamine by H_2_O_2_, in the presence of the separated, Cu^2+^ ion-functionalized C-dots and the 5′-end-amino-modified DBA (**1**), at identical concentrations of the constituents associated with the aptananozymes is shown in curve (f). A very inefficient oxidation rate of dopamine is observed. A linear relation between the oxidation rates as a function of the concentrations of dopamine is observed, consistent with a bimolecular reaction process in the homogeneous solution. Also, Figure 2B, curve (g) shows the rate of oxidation of dopamine at different concentrations of the substrate in the presence of Cu^2+^ ion-functionalized C-dots modified with a scrambled base sequence of the DBA (**1a**) that consists of the sequence (**1**). A substantially lower rate of oxidation of dopamine by the (**1a**)-modified Cu^2+^ ion-functionalized C-dots, as compared to the set of aptananozymes, is observed, yet is higher than the control system of separated constituents. The enhanced activity of the scrambled strands (**1a**)-modified C-dots as compared to the separated constituents is attributed to the electrostatic attraction of the protonated dopamine to the negatively charged (**1a**)-modified C-dots that results in a local concentration of the substrate close to the catalytic interface.

The rates of oxidation of dopamine by the set of aptananozymes reveal a dependence on the structure of the aptamer constituents linked to the Cu^2+^ ion-functionalized C-dots: (i) The aptananozyme composed of the 5′-end-DBA and catalyst, aptananozyme I, reveals a higher activity as compared to the 3′-end-DBA-modified catalyst, aptananozyme II. (ii) The activities of the 5′-end-DBA-modified aptananozymes are affected by the length of the spacer bridging the aptamer to the Cu^2+^ ion-functionalized C-dots. As the spacer bridging units increase from (TGTA) to (TGTA)_2_, the catalytic activity of the aptananozymes increases as compared to the aptananozyme composed of the DBA aptamer directly bound to the catalyst, aptananozyme I, following the order IV ≈ III > I. In contrast, the aptananozyme V composed of the longer spacer (TGTA)_3_ conjugated to the 5′-end-amino DBA aptamer reveals a lower catalytic activity as compared to aptananozyme I, leading to the order of aptananozymes activities IV ≈ III > I > V. The saturation curves corresponding to the rates of oxidation of dopamine at different dopamine concentrations were analyzed in terms of the Michaelis–Menten model, and the kinetic parameters of the set of aptananozymes and the control systems are summarized in Table 1. Specifically, the aptananozyme I reveals a 50-fold enhancement of catalytic activity as compared to the separated Cu^2+^ ion-functionalized C-dots/DBA constituent. Note that fitting the rates of the catalyzed oxidation of dopamine as a function of the substrate concentration according to the Michaelis–Menten model for the catalytic configurations of aptananozyme I, II, III, IV, and V leads to a best fit using a Hill coefficient of *n* ≈ 1. Thus, although four aptamer binding units are associated with the core catalytic nanoparticles, no cooperative effect between the different binding sites and the catalytic function is observed. This may be attributed to the low coverage of the Cu^2+^ ion-functionalized C-dots particles by the aptamers and the spatial separation of the binding aptamers that prohibits cooperative interactions (for a further discussion on the Hill coefficients of all aptananozymes, see the Supporting Information, Pages S6–S7). We find that the aptananozymes reveal a high stability (see Figure S8A and the accompanying discussion). Note that the catalytic activity of the DBA-modified Cu^2+^ ion-functionalized C-dots toward the oxidation of dopamine is selective for the Cu^2+^-modified particles, and other DBA-modified metal ion-functionalized C-dots such as Zn^2+^, Mn^2+^, Co^2+^, and Cd^2+^ ion-modified C-dots did not reveal any catalytic activity. The DBA-modified Fe^3+^ ion-functionalized C-dots revealed a catalytic activity toward the oxidation of dopamine, yet it was substantially lower as compared to that of the Cu^2+^ ion-functionalized C-dots, Figure S9A, Supporting Information. Note that the catalytic oxidation of dopamine by H_2_O_2_ in the presence of the aptamer-modified Cu^2+^ ion-functionalized C-dots is controlled by the loading of Cu^2+^ ions on the C-dots. (for further discussion and experimental results, see Figure S10).

**Table 1:** Kinetic Parameters Associated with the Aptananozymes I–V and Control Systems^a^

aptananozyme	*V*_max_ (μM min^–1^)	*K*_M_ (μM)	*k*_cat_ (10^–3^ s^–1^)	*k*_cat_/*K*_M_ (s^–1^ M^–1^)
IV	2.01 ± 0.1	181 ± 32	2.02 ± 0.1	11.2
III	1.97 ± 0.08	230 ± 31	1.98 ± 0.08	8.6
I	1.71 ± 0.04	232 ± 17	1.72 ± 0.04	7.4
II	1.52 ± 0.1	252 ± 57	1.53 ± 0.1	6.0
V	1.28 ± 0.05	324 ± 43	1.29 ± 0.05	4.0
scrambled	0.73 ± 0.04	464 ± 70	0.74 ± 0.04	1.6
separated^b^	0.04		0.04	

a All experiments were performed in a 5 mM MES buffer solution, pH 5.5, that included 5 mM MgCl_2_, 100 mM NaCl, and 0.2 μg·mL^–1^ of the respective aptananozymes or control system and 5 mM H_2_O_2_.

b The separated Cu^2+^ ion-functionalized C-dots/DBA system shows pseudo-first-order kinetics: *k* = 0.04 × 10^–3^ s^–1^.

Realizing that the composition of the Cu^2+^ ion-functionalized C-dots is identical for all aptananozymes, we searched for a possible origin for the different catalytic activities of the aptananozymes. Toward this goal, we examined the dissociation constants of the dopamine/aptananozyme complexes using ITC, and the derived dissociation constants are summarized in Table S2. (For the experimental binding curves leading to the derived *K*_d_ values and the respective control experiments, see Figure S11, panels i–vi, and the accompanying discussion).

From Table S2, we realize that the dissociation constant of the aptananozyme II is significantly higher than the *K*_d_ value of aptananozyme I. Thus, the lower binding affinity of the dopamine substrate to the aptamer units of aptananozyme II may account for the lower catalytic activity of aptananozyme II, as compared to that of aptananozyme I. The *K*_d_ values of the aptananozymes III and IV, are, however, very similar to the *K*_d_ value of aptananozyme I, and thus the binding affinities of dopamine to the aptamer binding sites cannot account for the enhanced catalytic activities of aptananozymes III and IV as compared to that of aptananozyme I. Presumably, the spacer units bridging the aptamer units to the catalytic Cu^2+^ ion-functionalized C-dots introduce a steric flexibility that allows an enhanced spatial proximity between the dopamine/DBA complex and the catalytic core that facilitates the oxidation of dopamine to aminochrome. The lower catalytic activity of longer-spaced aptananozyme, (TGTA)_3_, aptananozyme V, as compared to aptananozyme I, is, however, surprising in view of the flexibility anticipated for this aptananozyme. Nonetheless, the binding affinity of the aptananozyme V, Table S2, reveals a significantly higher dissociation constant (*K*_d_ = 3.9 ± 0.2 μM) as compared to that of aptananozymes I, III, and IV. The lower binding affinity of dopamine to aptananozyme V may well account for the lower catalytic activity of this aptananozyme. At present, the origin for the high *K*_d_ value of aptananozyme V is not known, yet it should be noted that all free, non-C-dots-modified DBA for aptananozymes I, II, III, IV, and V reveal very similar *K*_d_ values (*K*_d_ ≈ 1.09 μM).

**Figure 3:**
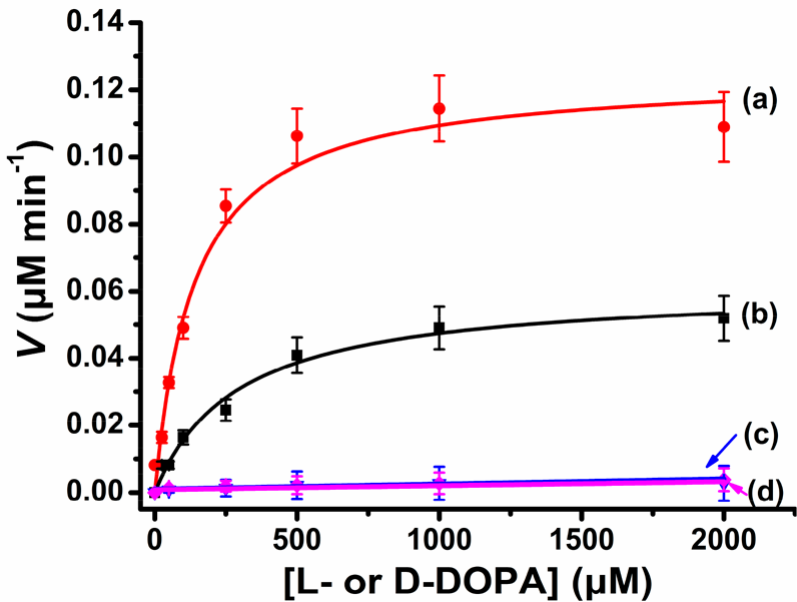
Rates corresponding to (a) the aptananozyme I-catalyzed oxidation of L-DOPA by H_2_O_2_ to generate L-dopachrome in the presence of variable concentrations of L-DOPA and (b) the aptananozyme I-catalyzed oxidation of D-DOPA by H_2_O_2_ to form D-dopachrome in the presence of variable concentrations of D-DOPA. (c, d) Rates corresponding to the oxidation of L-DOPA and D-DOPA in the presence of variable concentrations of L-/D-DOPA by H_2_O_2_, using the separated aptananozyme I and the aptamer (1), respectively. Error bars are derived from *N* = 3 experiments.

Besides the function of the aptamer units as binding sites for the concentration of the substrate at the catalytic core, the chiral features of the aptamer strands suggest that the aptananozymes could potentially induce chiroselective catalytic transformations. This feature was supported by the chiroselective oxidation of L- or D-DOPA (DOPA = 3,4-dihydroxyphenylalanine) by the aptananozyme I, composed of the Cu^2+^ ion-functionalized C-dots modified with the 5′-end-amino DBA (**1**), Figure 3. The diastereoisomeric discrimination between L-DOPA and D-DOPA by the nucleic acid aptamer led to a ca. two-fold enhanced oxidation of L-DOPA to L-dopachrome, Figure 3, curve (a), as compared to the oxidation of D-DOPA to D-dopachrome, curve (b). For comparison, curves (c) and (d) show the rates of oxidation of L-DOPA and D-DOPA in the presence of the separated Cu^2+^ ion-modified C-dots and the DBA (**1**), demonstrating the concentration effect of the aptananozyme on the oxidation of the L-/D-DOPA substrates. The catalytic oxidation of L-DOPA and D-DOPA by the aptananozyme I reveals Michaelis–Menten saturation curves, and the kinetic parameters corresponding to the respective oxidation processes are summarized in Table S3, Supporting Information. The enhanced catalytic performance of the aptananozyme I toward L-DOPA is attributed to the diastereoisomeric discrimination of L-DOPA and D-DOPA toward the aptamer that leads to a higher binding affinity of L-DOPA to the aptamer (*K*_d_ = 1.7 ± 0.4 μM) as compared to the lower binding affinity of D-DOPA to the aptamer (*K*_d_ = 6.6 ± 1 μM), Figure S11A and Table S4, Supporting Information.

**Figure 4:**
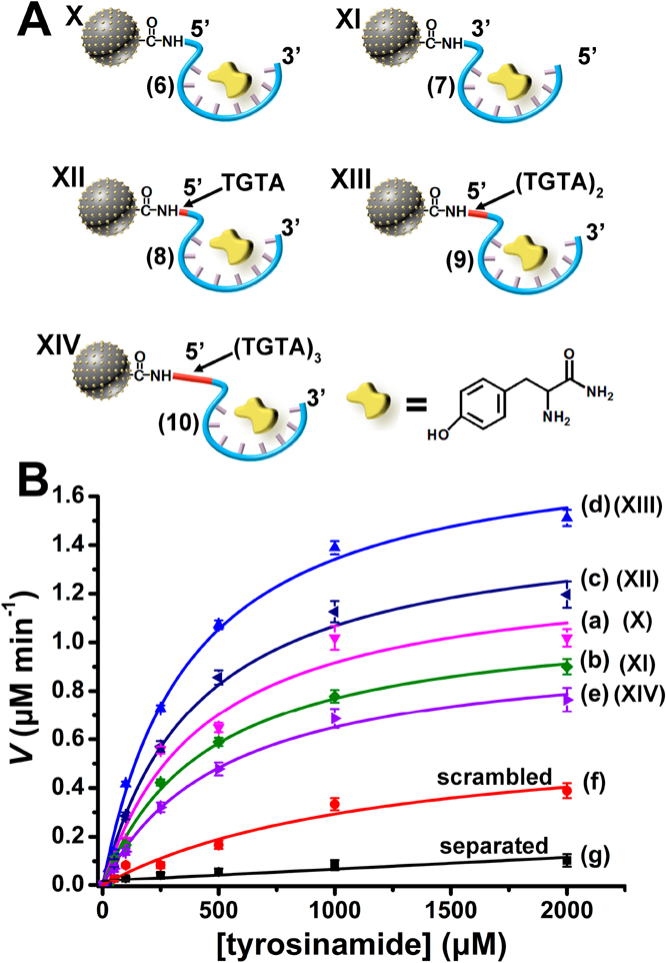
(A) The set of aptananozymes used for the catalyzed oxidation of l-tyrosinamide to amidodopachrome. (For the detailed sequences of the TBA conjugated to the Cu^2+^ ion-functionalized C-dots see the Experimental Section.) (B) Rates of oxidation of l-tyrosinamide to amidodopachrome by H_2_O_2_ using variable concentrations of l-tyrosinamide in the presence of the set of aptananozymes and the respective control systems. Error bars derived from *N* = 3 experiments.

To demonstrate the versatility of the aptananozyme concept, we synthesized a second set of Cu^2+^ ion-functionalized C-dots modified with the l-tyrosinamide binding aptamer (TBA) for the catalyzed oxygen insertion into the C–H bond of the l-tyrosinamide substrate to yield the catechol product that is further oxidized to amidodopachrome, Figure 1B. The set of aptananozymes is schematically presented in Figure 4A and includes the 5′-end-amino TBA directly linked to the Cu^2+^ ion-functionalized C-dots, aptananozyme X, the 3′-end-amino TBA directly linked to the Cu^2+^ ion-functionalized C-dots, aptananozyme XI, the 5′-end-amino TBA conjugated to the Cu^2+^ ion-functionalized C-dots through a (TGTA) spacer unit, aptananozyme XII, the 5′-end-amino TBA linked to the Cu^2+^ ion-functionalized C-dots through a (TGTA)_2_ spacer, aptananozyme XIII, and the 5′-end-amino TBA linked to the Cu^2+^ ion-functionalized C-dots through a (TGTA)_3_ spacer bridging unit, aptananozyme XIV. This set of aptananozymes was prepared, as before, by the coupling of the respective amino-modified TBA to the carboxylic acid functionalities associated with the C-dots using EDC/sulfo-NHS as coupling reagents that form the respective amide covalent-bond links. (The sequences of the different TBA strands are detailed in the Experimental Section.) The ICP-MS analyses indicated a loading of Cu^2+^ ions corresponding to 40 ± 3 μg per mg of C-dots, and spectroscopic measurements indicated an average loading of 4.3 ± 0.2 TBA units per single C-dots (see Table S5, Supporting Information). As before, the catalytic activities of the set of aptananozymes were compared to the activities of separated Cu^2+^ ion-functionalized C-dots and TBA (**6**), under the same experimental conditions, and to the Cu^2+^ ion-functionalized C-dots modified with the scrambled base-sequence (**6a**) comprising the TBA (**6**). Note that the scrambled strands associated with the C-dots reveals a residual small catalytic activity as compared to the separated system. This is because l-tyrosinamide is protonated and positively charged at experimental conditions, pH = 7.2, and thus electrostatically attracted and concentrated at the catalytic interface by the negatively charged scrambled strand.

The set of aptananozymes was applied as heterogeneous nanozymes for the oxygenation of l-tyrosinamide to the catechol product and subsequent oxidation to amidodopachrome, by the “magic” reaction mixture consisting of H_2_O_2_ and ascorbate (AA^–^). Treatment of l-tyrosinamide in the presence of the set of aptananozyme catalysts and the H_2_O_2_/AA^–^ mixture led to the formation of amidodopachrome that was spectroscopically followed at λ = 475 nm, Figure S12. The oxidation product suggests that the l-tyrosinamide substrate was transformed to the catechol product that was further oxidized to amidodopachrome. Control experiments indicated that no oxidation of l-tyrosinamide to amidodopachrome occurred in the presence of only H_2_O_2_ or only AA^–^, indicating that the mixture of the constituents is essential to drive the oxidation of l-tyrosinamide (Figure S13). Figure 4B, curves (a) to (e), depicts the rates of l-tyrosinamide oxidation to amidodopachrome, as a function of the l-tyrosinamide concentrations, using H_2_O_2_/AA^–^ as an oxidizing mixture, in the presence of the set of aptananozymes X–XIV, the scrambled and the separated TBA with Cu^2+^ ion-functionalized C-dots (for the time-dependent oxidation of the l-tyrosinamide substrates in the presence of variable substrate concentrations by the different aptananozymes and control systems, see Figure S14). For comparison, the rate of oxidation of l-tyrosinamide at different concentrations of l-tyrosinamide by the Cu^2+^ ion-functionalized C-dots modified with a strand (**6a**) consisting of the scrambled bases of TBA (**6**) are presented in Figure 4B, curve (f). In addition, the rate of oxidation of l-tyrosinamide at different concentrations of l-tyrosinamide in the presence of the separated Cu^2+^ ion-functionalized C-dots and the TBA (**6**) are displayed in Figure 4B, curve (g). The results demonstrate superior catalytic activities by the set of aptananozymes X–XIV as compared to the negligible catalytic activity revealed by the separated Cu^2+^ ion-functionalized C-dots and TBA constituents, curve (g). The catalytic activities of the aptananozymes relate to the structure of the TBA conjugated to the Cu^2+^ ion-functionalized C-dots. The catalytic features of the l-tyrosinamide oxygenation aptananozymes reveal some similarities to the catalytic functions of dopamine aptananozymes. That is, the linkage of the 5′-end of the TBA to the Cu^2+^ ion-functionalized C-dots, aptananozyme X, yields an aptananozyme of enhanced catalytic activity as compared to aptananozyme XI, where the 3′-end TBA is conjugated to the Cu^2+^ ion-functionalized C-dots. Similarly, the conjugation of the 5′-end TBA to the C-dots through spacers consisting of (TGTA) and (TGTA)_2_ bridges led to aptananozymes of enhanced catalytic performance XIII > XII, yet the longest spacer linking the 5′-end TBA to the Cu^2+^ ion-functionalized C-dots through the (TGTA)_3_ bridge, configuration XIV, yields the aptananozyme of the lowest catalytic activity. The order of catalytic activities of the l-tyrosinamide oxidizing aptananozymes is XIII > XII > X > XI > XIV. The entire set of l-tyrosinamide aptananozymes reveals Michaelis–Menten saturation curves that fit the experimental data with a Hill coefficient *n* ≈ 1. Thus, although four aptamer strands are associated with the catalytic core, no cooperative interactions between the aptamer binding domains exist. This result is attributed to the low coverage of the TBA units on the catalytic core. The Michaelis–Menten kinetic parameters derived for the different l-tyrosinamide aptananozymes are summarized in Table 2; the aptananozyme XIII reveals a 15-fold enhanced catalytic activity as compared to the separated Cu^2+^ ion-functionalized C-dots/TBA constituents. The oxidation of l-tyrosinamide to amidodopachrome in the presence of the H_2_O_2_/AA^–^ mixture is selective to the Cu^2+^ ion-functionalized C-dots, and other metal ion-functionalized C-dots (Zn^2+^, Co^2+^, Mn^2+^, Cd^2+^, and Fe^3+^) did not show any catalytic activities, Figure S9B. (For further discussion on the stability of the aptananozymes, see Figure S8B and accompanying discussion.) Note that the functionalization of the C-dots with Cu^2+^ ions is essential to drive the catalytic oxidation of dopamine or tyrosinamide, and bare C-dots did not show any catalytic performance (For further discussion demonstrating the selective oxygenation and oxidation of tyrosinamide, see Figure S15 and the accompanying discussion).

**Table 2:** Kinetic Parameters Corresponding to the Aptananozymes X–XIV and Control Systems^a^

	aptananozyme	*V*_max_ (μM min^–1^)	*K*_M_ (μM)	*k*_cat_ (10^–4^ s^–1^)	*k*_cat_/*K*_M_ (s^–1^ M^–1^)
	XIII	1.84 ± 0.08	375 ± 46	7.4 ± 0.2	1.97
	XII	1.51 ± 0.1	415 ± 71	6.1 ± 0.3	1.5
	X	1.32 ± 0.1	436 ± 62	5.3 ± 0.3	1.2
	XI	1.12 ± 0.06	460 ± 64	4.5 ± 0.2	0.98
	XIV	0.99 ± 0.05	531 ± 67	4.0 ± 0.1	0.75
	scrambled	0.6 ± 0.1	1202 ± 201	2.4 ± 0.3	0.2
	separated^b^	0.12		0.48	

a All experiments were performed in a 50 mM phosphate buffer solution at pH 7.2, containing 100 mM NaCl and 5 mM MgCl_2_, in the presence of the respective aptananozymes (0.5 μg mL^–1^) or control catalyst and 5 mM H_2_O_2_, 5 mM AA^–^.

b The separated Cu^2+^ ion-functionalized C-dots/TBA system shows pseudo-first-order kinetics: *k* = 0.48 × 10^–4^ s^–1^.

Table S6 summarizes the ITC-evaluated dissociation constants of the series of aptananozymes X-XIV. (For the experimental binding curves leading to the *K*_d_ values, derived from the binding curves and the respective control experiments, see Figure S16, panels i–vi, and the accompanying discussion.) The slightly lower catalytic activity of aptananozyme XI as compared to the catalytic activity of aptananozyme X can be attributed to the lower binding affinity of the l-tyrosinamide substrate to the aptananozyme XI (*K*_d_ (XI) = 1.24 ± 0.05 μM vs *K*_d_ (X) = 0.83 ± 0.01 μM). The *K*_d_ values of the aptananozymes X, XII, and XIII are very similar, indicating similar binding affinities toward l-tyrosinamide. Thus, the enhanced catalytic features of the aptananozymes XII and XIII are attributed, as before, to the enhanced flexibility of the (TGTA)- and (TGTA)_2_-spaced aptamers that lead to a spatial proximity between the l-tyrosinamide/TBA complexes and the catalyst Cu^2+^ ion-functionalized C-dots surface. As before, the long spaced (TGTA)_3_ aptananozyme, XIV, reveals a lower catalytic activity as compared to the reference aptananozyme X, despite the flexibility of the aptamer binding site. Also, in this case, we find that the dissociation constant of aptananozyme XIV is higher than the *K*_d_ value of the reference aptananozyme X (*K*_d_ (XIV) = 1.4 ± 0.1 μM vs K_d_ (X) = 0.83 ± 0.01 μM) (For further experiments addressing the enantioselective oxidation of L-/d-tyrosine by the aptananozyme X, see Figure S17 and the accompanying discussion, Supporting Information).

**Figure 5:**
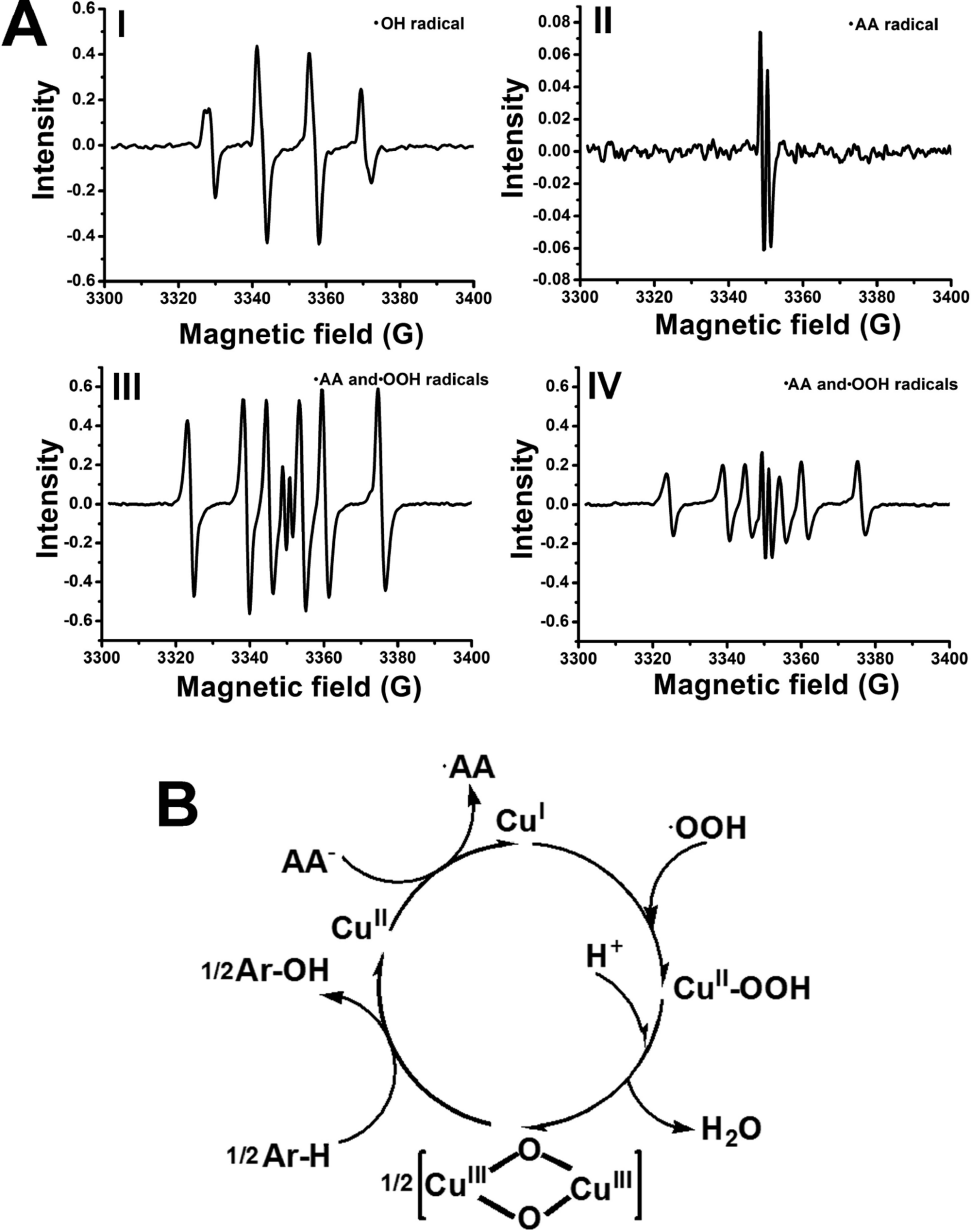
(A) ESR spectra corresponding to Panel I, the ·OH generated by the aptananozyme X in the presence of H_2_O_2_. Panel II, the ·AA radical generated by the aptananozyme X in the presence of ascorbate. Panel III, the mixture of ·AA and peroxy radical ·OOH generated by the aptananozyme X in the presence of the mixture of AA^–^/H_2_O_2_. Panel IV, the mixture of ·AA and ·OOH generated by the aptananozyme X in the presence of the mixture of AA^–^/H_2_O_2_ with added l-tyrosinamide. (B) Schematic suggested mechanistic cycle for the oxygenation of the Ar–H bond of l-tyrosinamide to catechol product by the Cu^II^-functionalized C-dots aptananozymes.

Finally, we addressed mechanistic aspects related to the reactive species generated by the Cu^2+^ ion-functionalized C-dots in the presence of H_2_O_2_ and, particularly, in the presence of the H_2_O_2_/AA^–^ (ascorbate) mixture. In fact, Cu^2+^ enzymes or Fe^3+^ enzymes, such as catechol oxidase or cytochrome P-450, and methane monooxygenase act in nature as biocatalysts oxidizing catechol derivatives to quinones or biocatalysts mediating C–H bond oxygenation, and the native mechanisms associated with the biocatalysts attracted substantial research efforts.^47^ The aptamer-modified Cu^2+^-functionalized C-dots could, thus, be considered as nanozymes emulating these biological processes. Accordingly, we evaluated by electron-spin resonance (ESR) spectroscopy the formation of possible reactive oxygen species generated by the Cu^2+^-modified C-dots in the presence of H_2_O_2_ or H_2_O_2_/AA^–^, the reaction mixtures used in the catalyzed oxidation of dopamine to aminochrome or the C–H bond oxygenation of the l-tyrosinamide, respectively, Figure 5A. Subjecting the DBA-modified Cu^2+^ ion-functionalized C-dots to H_2_O_2_ yields the hydroxyl radical (·OH) as a reactive oxygen species, Figure S18. A treatment of the TBA-modified Cu^2+^ ion-functionalized C-dots with an aqueous H_2_O_2_ solution yields, similarly, to the formation of the hydroxyl radical (·OH), Figure 5A, Panel I. Subjecting the aptananozyme consisting of TBA-modified Cu^2+^ ion-functionalized C-dots to an AA^–^ solution (in the absence of H_2_O_2_) yields to the formation of the ascorbate radical, AA·, Figure 5A, Panel II. Subjecting the TBA-modified Cu^2+^ ion-functionalized C-dots aptananozyme to the mixture of H_2_O_2_ and AA^–^, leads, however, to the formation of the mixture of ascorbate radical, AA·, and peroxy-radical ·OOH without the formation of the ·OH species, Figure 5A, Panel III. These results suggest that the reactive species in the oxidation of dopamine is the hydroxyl radical, ·OH, whereas the mixture of AA· and ·OOH species generated by the TBA-modified Cu^2+^ ion-functionalized C-dots in the AA^–^/H_2_O_2_ solution participate in the C–H bond oxygenation of l-tyrosinamide. These results imply that the formation of the ·OOH is intimately related to the formation of the AA· species. The addition of the l-tyrosinamide substrate to the mixture of H_2_O_2_/AA^–^ leads, in the presence of the added TBA-modified Cu^2+^ ion-functionalized C-dots aptananozyme, to the formation of the two intermediates AA· and ·OOH species, Figure 5A, Panel IV, yet the content of the ·OOH is dampened while the content of the AA· in the reaction mixture is almost unaffected. These results indicate that the ·OOH is a reactive species in the C–H bond oxygenation. A possible mechanistic path for the ·OH-stimulated oxidation of the catechol dopamine residue to the quinone product is outlined in Figure S19. On the basis of previous reports suggesting the peroxy radical mediated the formation of bis-μ-oxo dicopper species in analogy to tyrosinase,^48^ we formulate the cycle presented in Figure 5B as a possible path for the oxygenation of the l-tyrosinamide aryl-H (Ar–H) bond into the catechol product. The set of reactions summarized in Figure S20, and the accompanying discussion, suggests a possible route for the formation of ·OOH by the Cu^2+^ ion-functionalized C-dots, in the presence of the H_2_O_2_/AA^–^ mixture. In fact, XPS measurements indicated the presence of Cu^III^ species generated upon treatment of the Cu^II^-functionalized C-dots with the H_2_O_2_/AA^–^ mixture, Figure S21, supporting the formation of the Cu^III^-μ-oxo-bridged intermediate. Also, no reactive oxygen species on the C-dots could be detected.

## Conclusions

In conclusion, the present study has introduced the concept of aptananozymes, where sequence-specific aptamer strands are conjugated to nanosized heterogeneous catalysts as a means to enhance the catalytic activities of nanozymes. The binding of the reaction substrate at the catalyst core by means of aptamer-substrate complexes provides a means to concentrate the substrate in proximity to the catalytic interface, in analogy to the functions of native enzymes. This concept has been demonstrated by developing aptananozymes that catalyze the oxidation of dopamine to aminochrome and the oxygen insertion into aryl-H bonds of l-tyrosinamide to yield a catechol product that is further oxidized to amidodopachrome. By the structured engineering of the aptamer sequences, controlled catalytic functions of the aptananozymes were demonstrated. Note that, in contrast to previous reports demonstrating the similar oxidation process by homogeneous metal–organic complex-aptamer conjugates (nucleoapzymes),^43^ the present study extended the concept to heterogeneous catalysts. The present study introduces an important “missing” link into the area of hybrid catalytic nucleic acid nanostructures. The concept complements the “nucleoapzyme” and “photonucleoapzyme” paradigms where homogeneous catalysts or photosensitizers were conjugated to aptamer sequences to yield superior enzyme-like catalysts and photocatalysts. The approach introduced in this study paves a way for a versatile method to develop diverse aptananozymes by conjugating aptamers to other catalytic nanoparticles and, particularly, to conjugate aptamers to other metal-ion modified C-dots to drive other catalytic reactions. Furthermore, the study indicated the significance of bridging spacer units on the catalytic efficacy and substrate binding affinities of the aptananozymes. Further studies should be directed, however, to evaluate the effects of additional spacer lengths and base compositions on the performance of the aptananozymes. Particularly, the introduction of stimuli-responsive reconfigurable spacers, such as G-quadruplexes or light-responsive bridges, would be interesting to switch the catalytic activities of the aptananozymes.

## Experimental Section

The C-dots were prepared according to the previously reported method.^44^

Sequences used in the study were commercially ordered (Integrated DNA Technologies, IDT):

(**1**) Amino-DBA for 5′-linked aptananozyme (I): 5′-NH_2_–CGACGCCAGTTTGAAGGTTCGTTCGCAGGTGTGGAGTGACGTCG-3′.

(**1a**) Amino-scrambled DBA for 5′-linked aptananozyme: 5′-NH_2_-GACTAGCGTGTGTGATGGGACCTTAGGCCGTCACGGGGCTTAGT-3′.

(**2**) Amino-DBA for 3′-linked aptananozyme (II): CGACGCCAGTTTGAAGGTTCGTTCGCAGGTGTGGAGTGACGTCG-NH_2_-3′.

(**3**) Amino-DBA with (TGTA) spacer for 5′-linked aptananozyme (III): 5′-NH_2_-TGTA-CGACGCCAGTTTGAAGGTTCGTTCGCAGGTGTGGAGTGACGTCG-3′.

(**4**) Amino-DBA with (TGTA)_2_ spacer for 5′-linked aptananozyme (IV): 5′-NH_2_-TGTATGTA-CGACGCCAGTTTGAAGGTTCGTTCGCAGGTGTGGAGTGACGTCG-3′.

(**5**) Amino-DBA with (TGTA)_3_ spacer for 5′-linked aptananozyme (V): 5′-NH_2_-TGTATGTATGTA-CGACGCCAGTTTGAAGGTTCGTTCGCAGGTGTGGAGTGACGTCG-3′.

(**6**) Amino-TBA for 5′-linked aptananozyme (X): 5′-NH_2_-TGTGGTGTGTGAGTGCGGTGCCC-3′.

(**6a**) Amino-scrambled TBA for 5′-linked aptananozyme: 5′-NH_2_-TGTGGTGTGTGAGTGCGGTGCCC-3′.

(**7**) Amino-TBA for 3′-linked aptananozyme (XI): TGTGGTGTGTGAGTGCGGTGCCC-NH_2_-3′.

(**8**) Amino-TBA with (TGTA) spacer for 5′-linked aptananozyme (XII): 5′-NH_2_-TGTA-TGTGGTGTGTGAGTGCGGTGCCC-3′.

(**9**) Amino-TBA with (TGTA)_2_ spacer for 5′-linked aptananozyme (XIII): 5′-NH_2_-TGTATGTA-TGTGGTGTGTGAGTGCGGTGCCC-3′.

(**10**) Amino-TBA with (TGTA)_3_ spacer for 5′-linked aptananozyme (XIV): 5′-NH_2_-TGTATGTATGTA-TGTGGTGTGTGAGTGCGGTGCCC-3′.

For the details of the conjugation of the aptamers to the C-dots, the characterization of the aptamer and C-dots conjugates, and the kinetic measurements see Supporting Information Pages S2–S5.

## Supporting Information Available

Experimental procedures, quantification of aptamer and Cu^2+^ ion-functionalized C-dots, TEM image, XPS measurements, FTIR spectra, time-dependent absorbance changes upon the oxidation of different concentrations of dopamine or l-tyrosinamide, ITC measurements (PDF)
