# Supplementary material for: Aptamer-Modified Cu2+-Functionalized C-Dots: Versatile Means to Improve Nanozyme Activities-“Aptananozymes”
Source: J Am Chem Soc. 2021 Jul 21;143(30):11510–9. doi: 10.1021/jacs.1c03939 (PMC8856595; doi:10.1021/jacs.1c03939)
Supplement: Supplementary file 1 — ja1c03939_si_001.pdf [file ja1c03939_si_001.pdf]

Supporting Information

Aptamer-Modified Cu^{2+}-Functionalized C-Dots: Versatile Means to Improve Nanozymes Activities- "Aptananozymes"

Yu Ouyang[†a], Yonatan Biniuri[†a], Michael Fadeev[a], Pu Zhang[a], Raanan Carmieli [b], Margarita Vázquez-González[a], Itamar Willner[*a]

a. The Institute of Chemistry, The Hebrew University of Jerusalem, Jerusalem 91904, Israel.

b. Department of Chemical Research Support, Weizmann Institute of Science, Rehovot, 76100, Israel.

[†]These authors contributed equally.

E-mail: Itamar.willner@mail.huji.ac.il

Instruments

Absorption spectra were recorded on a UV-2450 spectrophotometer (UV, Shimadzu). Kinetic measurements were performed at 25 °C using a Biotek Synergy H1 microplate reader, equipped with a Biotek dual dispensing unit, and using Corning 3696 96-hald-well plates. Dissociation constants were evaluated using Isothermal titration calorimetry (ITC) instrument (Malvern instruments MicroCal PEAQ-ITC). X-ray photoelectron spectroscopy (XPS) measurements were performed with an Axis Ultra photoelectron spectrometer from Kratos Analytical. Fourier-transform infrared spectroscopy (FTIR) measurements were performed using a Nicolet iS50 FTIR Spectrometer. Transmission Electron Microscope was performed on a Tecnai G2 Spirit TWIN T12. Cu^{2+}-ions-functionalized C-dots were characterized by coupled plasma mass spectrometry using an Inductively Coupled Plasma-Mass Spectrometry (ICP) instrument. Electron spin resonance (ESR) measurements were carried out at room temperature using a Bruker ELEXYS E500 spectrometer operating at X-band frequencies (9.5 GHz) and a Bruker ER4102ST resonator.

Preparation of C-dots and their modification with Cu^{2+}-ions

The C-dots were synthesized according to Wang *et al*[1]. Citric acid and urea were mixed in water and heated for 4-5 min in a domestic 750 W microwave. This solid was then transferred to a vacuum oven and heated at 60℃ for 1 h to remove the residual small molecules. An aqueous solution of the C-dots was purified in a centrifuge (3000 g, 20 min) to remove large or agglomerated particles. The resulting colored (brown) aqueous solution remained indefinitely stable at various concentrations. The obtained

C-dots solution was purified by 3 kDa and 10 kDa cutoff filters under 9168 g for 30 min, respectively. Finally, the C-dots solution of below 3 kDa was frozen by liquid nitrogen and dried through lyophilization.

The Cu^{2+} ions-functionalized C-dots were prepared as follows: copper (II) chloride (0.01 M) and C-dots (2 mg) were mixed in solution of 1.8 mL ethanol (96%, *v*/*v*) and 200 uL H_2O. After shaking overnight, the mixture was under centrifugation at 10000 rpm for 5 min to collect the sediment. The sediment was washed with solution of 1.8 mL ethanol (96%, *v*/*v*) and 200 uL H_2O for 4 times, and then dried.

Modification of Cu^{2+}-ions-functionalized C-dots with amino-modified aptamers or amino-functionalized control strands

A 50 uL solution of Cu^{2+}-ions-functionalized C-dots (4 mg mL^{-1}) were subsequently added to equal volumes of 20 uL EDC (50 mM) and 50 uL sulfo-NHS (50 mM) in MES buffer (5 mM, pH 5.5) and left to react for 15 min. The above mixture was sequentially added 100 μL of 100 mM phosphate buffer, pH 7.2 and 100 μL of amino-functionalized aptamer (200 μM in 100 mM phosphate buffer, pH 7.2). The coupling reaction was performed at room temperature for 12 h. The solution after the reaction was purified by 10 kDa cutoff filters under 10000 g for 30 min and repeat for 4 times.

Evaluation of the loading of the aptamers on Cu^{2+}-ions-functionalized C-dots

After loading the amino-functionalized aptamers to the Cu^{2+}-ions-functionalized C-dots, the concentration of the Cu^{2+}-ions was determined by the ICP. Following the same procedure of preparation of attaching the aptamers to Cu^{2+}-ions-functionalized C-

dots, the ratios of C-dots to Cu^{2+}-ions are very similar to each other (1 : 0.04, mg mL^{-1}).

The ratios between the Cu^{2+}-ions-functionalized C-dots and aptamer could be determined by the relative calibration curves. As we can see from the Figure S5A and B, the two calibration curves at 260 and 338 nm were formed by the different concentrations of the Cu^{2+}-modified C-dots solution by UV spectra. Figure S5C and D show the UV spectra of 5′-DBA and 5′-TBA associated with Cu^{2+}-ions-functionalized C-dots. After we attach the NH_2-aptamers to the C-dots, the calibration curve at 330 nm is required to quantify the amount of C-dots, while the absorbance of C-dots at 260 nm is obtained. By the subtraction of the spectrum at 260 nm, the net absorbance of the nucleic acids associated with the C-dots is evaluated. It should be noted that multiple groups of experiments were executed to achieve the similar ratio of Cu^{2+}-ions/aptamers to C-dots.

Kinetic measurements with aptananozymes

Kinetic measurements were performed at 25 °C using a Biotek Synergy H1 microplate reader equipped with a Biotek dual dispensing unit and using Corning 3696 96-well plates. For dopamine oxidation, the aptananozymes (0.2 μg mL^{-1}) were dissolved in 5 mM MES buffer, pH 5.5, 5 mM $MgCl_2$, 100 mM NaCl, and 10 μL of dopamine, consisting of variable concentrations which were added to the respective wells. Subsequently, 10 μL of H_2O_2 (final concentration 1 mM) was dispensed into each well, and the absorbance values of the oxidized products (absorbance at 480 nm, ε = 3058 M^{-1} cm^{-1}) were measured in the different wells for a time interval of 60 min. For the L-tyrosinamide oxidation process, the aptananozymes (0.5 μg mL^{-1}) were dissolved

in in 50 mM phosphate buffer solution (pH 7.2, containing 100 mM NaCl and 5 mM $MgCl_2$), and 10 μL of L-tyrosinamide, consisting of variable concentrations which were added to the respective wells. Subsequently, 10 μL of H_2O_2 (final concentration 5 mM) was dispensed into each well, and the absorbance values of oxidized products (absorbance at 475 nm, $\varepsilon = 3600\ M^{-1}\ cm^{-1}$) were measured in the different wells for a time interval of 120 min. As for the L-DOPA and D-DOPA oxidation process, the aptananozyme I (0.2 ug mL^{-1}) was dissolved in in 50 mM phosphate buffer solution (pH 7.2, containing 100 mM NaCl and 5 mM $MgCl_2$), and 10 μL of L-tyrosinamide, consisting of variable concentrations which were added to the respective wells. Subsequently, 10 μL of H_2O_2 (final concentration 1 mM) was dispensed into each well, and the absorbance values of oxidized products (absorbance at 475 nm, $\varepsilon = 3600\ M^{-1}$ cm^{-1}) were measured in the different wells for a time interval of 120 min.

ESR measurements.

Radical species such as ·OH and AA·, and ·OOH , were detected using the ESR spin trapping technique coupled with a spin trap 3,4-dihydro-2-methyl-1,1-dimethylethyl ester-2H-pyrrole-2-carboxylic acid-1-oxide (BMPO). Typically, five mixtures of H_2O_2 (5 mM) and aptananozyme I (0.2 μg mL^{-1}), H_2O_2 (5 mM) and aptananozyme X (0.5 μg mL^{-1}), ascorbic acid (5 mM) and aptananozyme X (0.5 μg mL^{-1}), or H_2O_2 (5 mM) and ascorbic acid (5 mM) and aptananozyme X (0.5 μg mL^{-1}), H_2O_2 (5 mM) and ascorbic acid (5 mM) and aptananozyme X (0.5 μg mL^{-1}) and L-tyrosinamide (1 mM) were prepared in phosphate buffer solution (pH 7.2, containing 100 mM NaCl and 5 mM $MgCl_2$), including BMPO (0.01 M). The ESR measurements

were taken with the following conditions: Centerfield, 3352.00 G; Sweepwidth, 100.0 G; MW Power, 20.02 mW; Modulation Amplitude, 1.00 G; Modulation Frequency, 100.00 kHz; No of points 512; Conversion Time, 40.96 ms; Conversion Time, 40.96 ms.

ITC evaluation of the K_d values of dopamine or D-/L-DOPA bound to DBA-functionalized aptananozymes and L-tyrosinamide to TBA-functionalized aptananozymes

A stock solution of the respective aptananozyme, 20 μM, in a 5 mM MES buffer pH 5.5, 100 mM NaCl and 5 mM $MgCl_2$ for the DBA-aptananozymes or 50 mM PBS pH 7.2, 100 mM NaCl, 5 mM $MgCl_2$ for the TBA-aptananozyme were prepared. Stock solutions of dopamine (600 μM), L-DOPA (1 mM), D-DOPA (1 mM) and L-tyrosinamide (300 μM) in buffer solutions identical to those of the corresponding aptananozymes were prepared. Also, control experiment involving the mixing of the host-in-buffer and the gest-in-buffer in order to evaluate the fitness of the mixing enthalpies to the experiments. The respective aptananozymes were loaded into the sample cell, 280 μL, and the ITC instrument syringe was loaded with the respective buffer-matched ligand stock solution. The aptananozyme sample was titrated by injecting repeated 2 μL aliquots of the respective ligand into the measurement cell (total 15-18 injections). The heat difference between the measuring and reference cell, upon each injection were evaluated under the following conditions: Cell reference power set at 10 μW, syringe rotation rate set to 750 RPM, initial delay of 60 seconds before the first injection to ensure equilibration of sample cell, injections spaced at 150-300

seconds to ensure baseline stabilization between measurements. The C-value (C-value = [aptamer in cell]/K_d of free aptamer) were evaluated before the experiments and were in the range of 3-20 for all aptananozymes. Each binding experiment was repeated n = 2. The resulting K_d fitting was performed using the instrument (MicroCal PEAQ-ITC Analysis Software) with the "one set of sites" binding model and free parameters for K_d, Hill coefficient, and ΔH. The experimental ITC curves are provided in Figures S4, Figure S11, Figure S11 (A), and Figure S16. The derived K_d values are summarized in table S2, Table S4, and Table S6. All fitted K_d curves yielded a Hill coefficient of 0.9 to ~1, implying a 1:1 complex between the ligands and the aptamers.

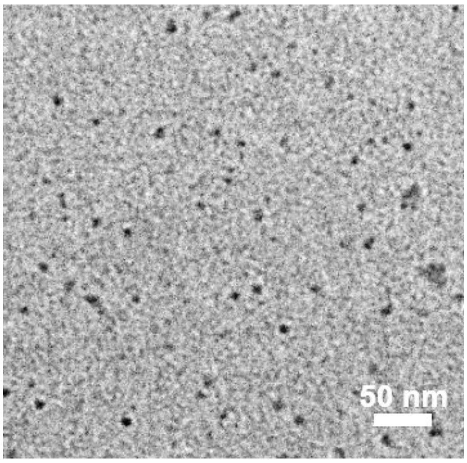

Figure S1. TEM image of C-dots.

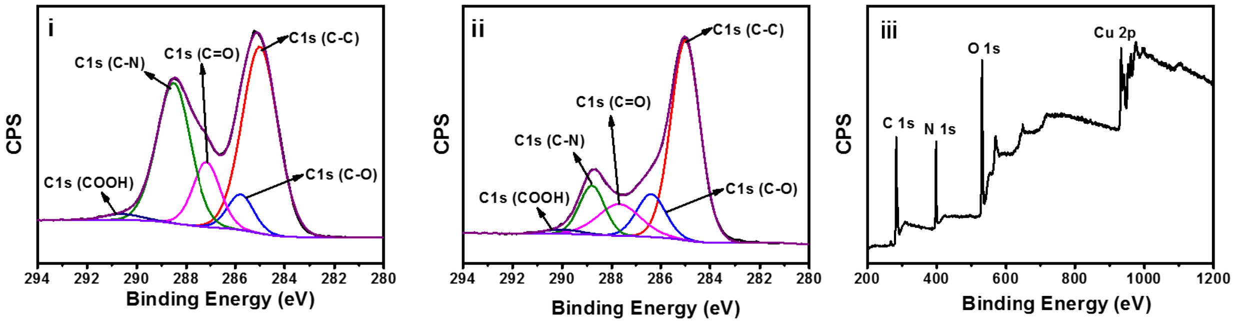

Figure S2. High-resolution XPS spectra of C (i, C-dots) and (ii, Cu^{2+}-ions-functionalized C-dots). (iii) Survey spectrum of Cu^{2+}-ions-functionalized C-dots.

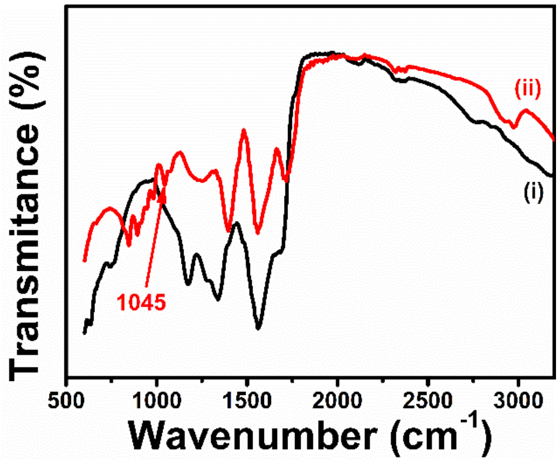

Figure S3. FTIR spectra of (i) C-dots, and (ii) Cu^{2+}-ions-functionalized C-dots.

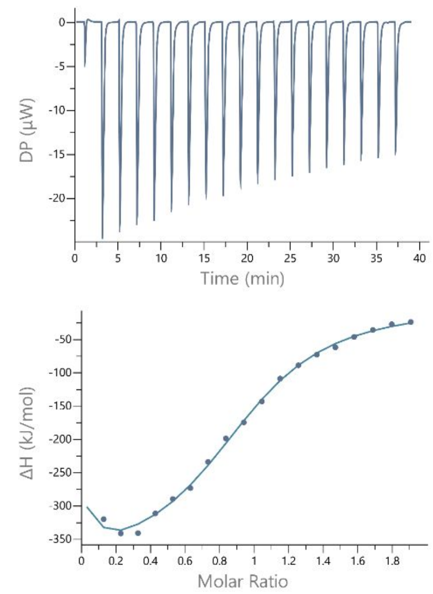

Figure S4. ITC plots (heat flow *vs* time and molar enthalpy change *vs* molar ratio) of C-dots and Cu^{2+}-ions in water.

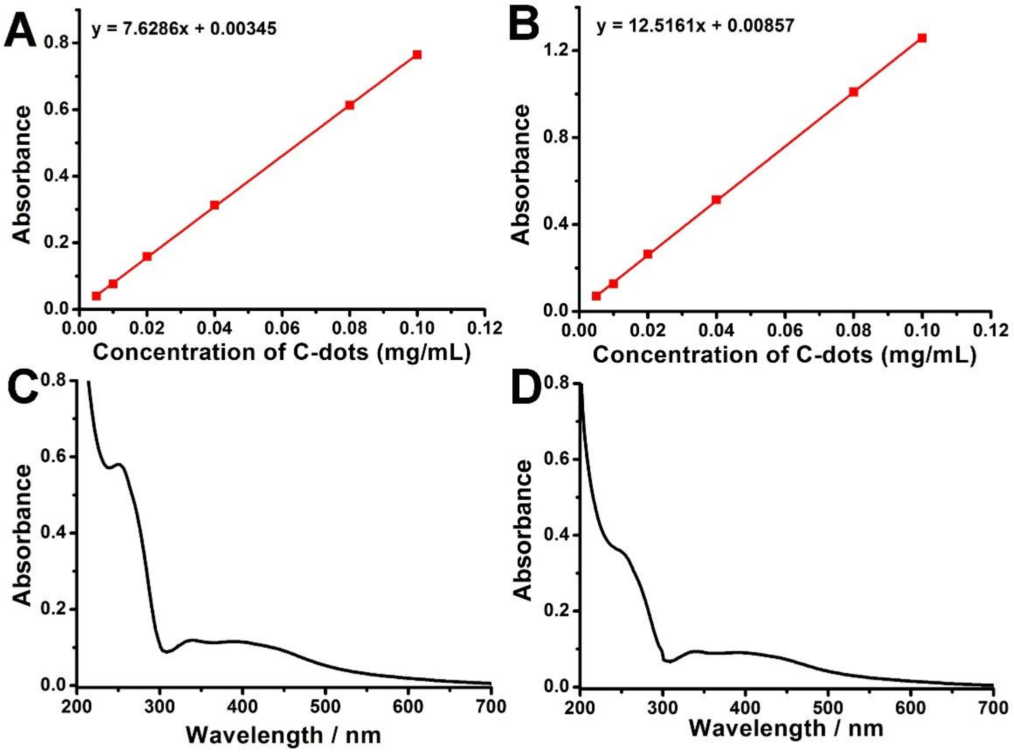

Figure S5. The calibration curves of Cu^{2+}-ions-functionalized C-dots at 338 nm (A) and 260 nm (B), respectively. (C) The UV curve of 5′-DBA associated with Cu^{2+}-ions-functionalized C-dots (aptananozyme I). (D) The UV curve of 5′-TBA associated with Cu^{2+}-ions-functionalized C-dots (aptananozyme X). (The way to evaluate aptamers on the C-dots see part of "Evaluation of the loading of the aptamers on Cu^{2+}-ions-functionalized C-dots.").

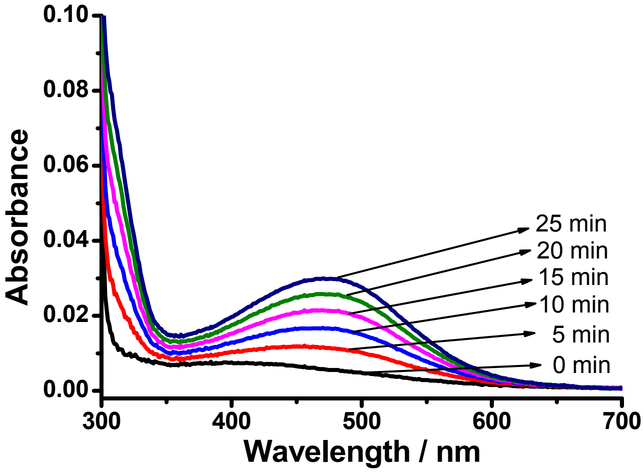

Figure S6. Time-dependent absorption spectra of oxidized dopamine in the presence of 0.2 µg mL^{-1}Aptananozyme I, 1 mM dopamine, 5 mM H_2O_2.

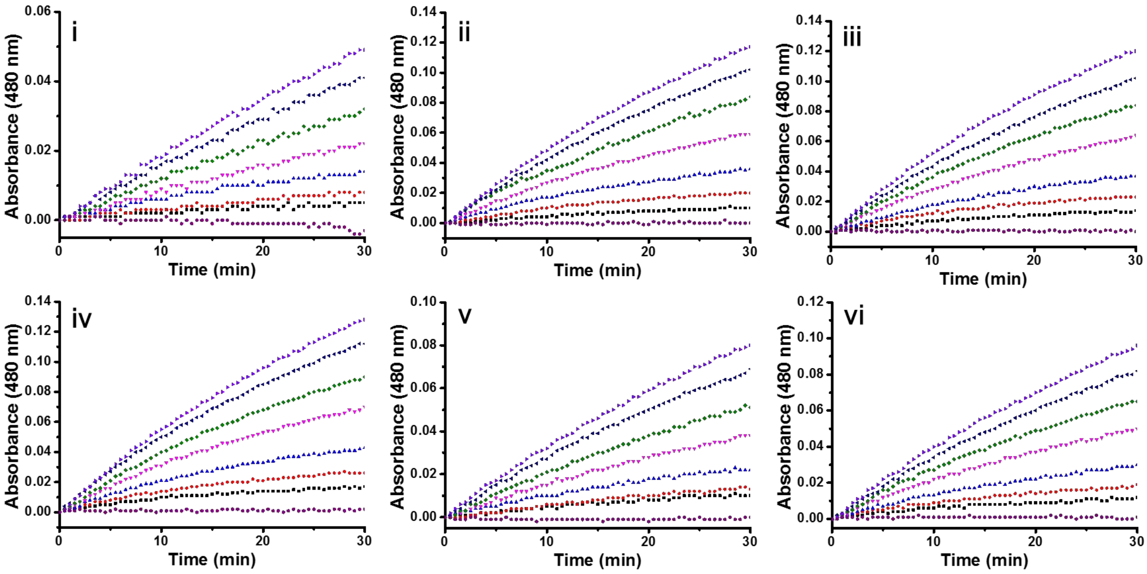

Figure S7. Time-dependent absorbance changes upon the oxidation of different concentrations of dopamine to aminochrome by the aptananozymes I-V: (a) 0, (b) 25, (c) 50, (d) 100, (e) 250, (f) 500, (g) 1000, (h) 2000 µM. (i) Cu^{2+}-ions-functionalized C-dots with scrambled DBA. (ii) Aptananozyme I. (iii) Aptananozyme III. (iv) Aptananozyme IV. (v) Aptananozyme V. (vi) Aptananozyme II. For all systems, the condition of the experiments corresponded to 0.2 µg mL^{-1} Aptananozymes, 5 mM H_2O_2.

Stabilities of the aptananozymes for the oxidation of dopamine and oxygenation of L-tyrosinamide.

Figure S8 shows studies addressing the stabilities of the aptananozymes catalyzing the oxidation of dopamine to aminochrome by H_2O_2, and the aptananozymes catalyzing

the oxygenation of L-tyrosinamide to the catechol product and subsequently to amidodopachrome in the presence of the H_2O_2/ascorbate mixture.

In these experiments, the aptananozyme IV or the aptananozyme XIII was subjected as catalyst for the oxidation of dopamine or oxygenation of L-tyrosinamide, respectively. The catalysts run for four catalytic cycles, where the particles were separated by centrifugation after each catalytic cycle. Figure S8A shows the rates of oxidation of dopamine to aminochrome at variable concentrations of dopamine following four catalytic cycles. Figure S8B shows the rates of oxidation of L-tyrosinamide to amidodopachrome at variable concentrations of L-tyrosinamide following four catalytic cycles. The catalytic rates are basically identical and the minute difference of V_{max} values. < 10 % are attributed to incomplete recovery of the catalytic particles upon centrifugation.

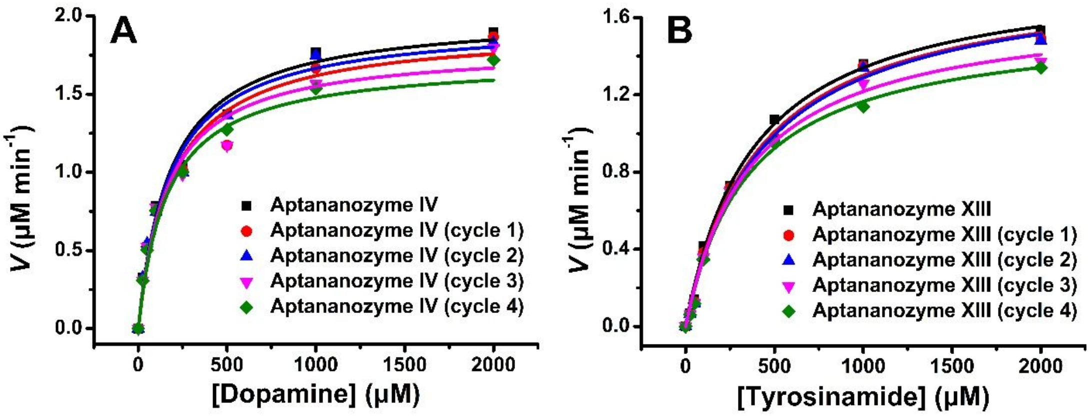

Figure S8. (A) Rates of oxidation of dopamine to aminochrome using variable concentrations of dopamine in the presence of aptananozyme IV, 0.2 µg mL^{-1}, H_2O_2, 5 mM following four times. (B) Rates of oxidation of L-tyrosinamide to amidodopachrome using variable concentrations of L-tyrosinamide in the presence of 0.5 µg mL^{-1} aptananozyme IV, 5 mM H_2O_2 and AA^-, following four times.

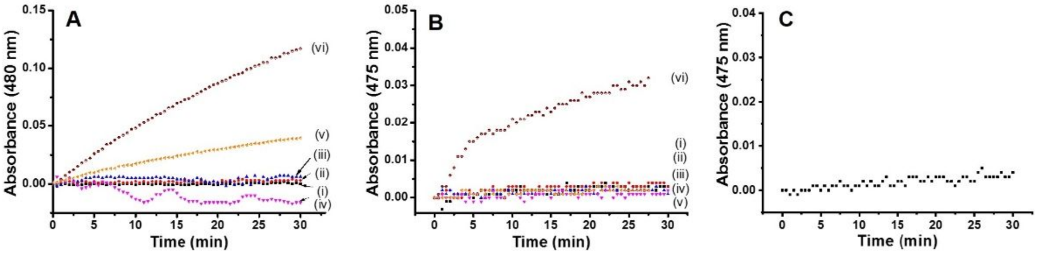

Figure S9. (A) Time-dependent absorbance changes upon the oxidation of dopamine to aminochrome by 5′-end-DBA-modified different metal-ions (i, Cd^{2+}; ii, Co^{2+}; iii, Mn^{2+}; iv, Zn^{2+}; v, Fe^{3+}; vi, Cu^{2+})-functionalized C-dots in presence of dopamine and H_2O_2. For all systems, the condition of the experiments corresponded to 0.2 μg mL^{-1} dopamine-aptananozymes, 5 mM H_2O_2. (B) Time-dependent absorbance changes upon the oxidation of L-tyrosinamide to amidodochrome by 5′-end-TBA-modified different metal-ions (i, Cd^{2+}; ii, Co^{2+}; iii, Mn^{2+}; iv, Zn^{2+}; v, Fe^{3+}; vi, Cu^{2+})-functionalized C-dots in presence of L-tyrosinamide, AA^{-}, and H_2O_2. For all systems, the condition of the experiments corresponded to 0.5 μg mL^{-1} L-tyrosinamide-aptananozymes, 5 mM H_2O_2, 5 mM AA^{-}. (C) Time-dependent absorbance changes upon the oxidation of L-tyrosinamide to amidodochrome by Cu^{2+}-ions (50 ng mL^{-1}) in presence of L-tyrosinamide, AA^{-}, and H_2O_2.

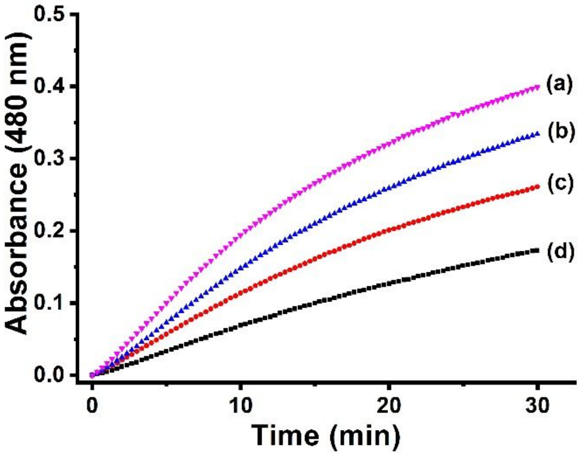

Figure S10 Oxidization of dopamine, 1 mM, by aptananozyme I (0.5 μg mL^{-1}) subjected to H_2O_2, 5 mM with different mass ratio between C-dots and Cu^{2+}-ions (a, 1:0.18; b, 1:0.13; a, 1:0.08; d, 1:0.04).

Figure S10 presents the raw heat plots (upper panels) and derived titration curves (lower panels) corresponding to (i) the scrambled DBA-aptananozyme and (ii)-(vi) the respective aptananozymes I-V.

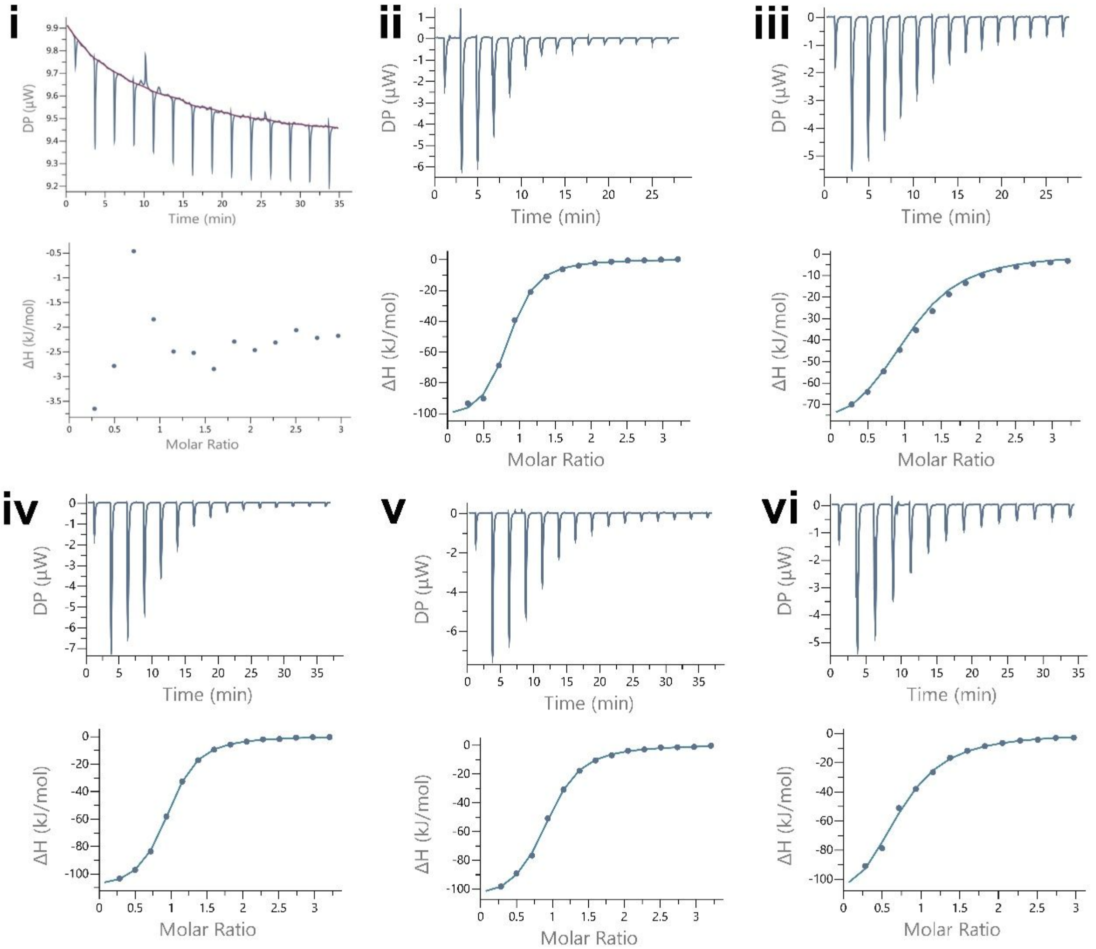

Figure S11. ITC plots (heat flow *vs* time and molar enthalpy change *vs* molar ratio) of different aptananozymes, with dopamine in 5 mM MES pH 5.5, 5 mM $MgCl_2$, 100 mM NaCl: (i) Cu^{2+}-functionalized C-dots with scrambled DBA. (ii) aptananozyme I. (iii) aptananozyme II. (iv) aptananozyme III. (v) aptananozyme IV. (vi) aptananozyme V.

The derived K_d values are summarized in Table S2.

Figure S11A presents the raw heat plots (upper panels) and derived titration curves (lower panels) corresponding to (i) L-DOPA-aptananozyme and (ii) D-DOPA-aptananozyme.

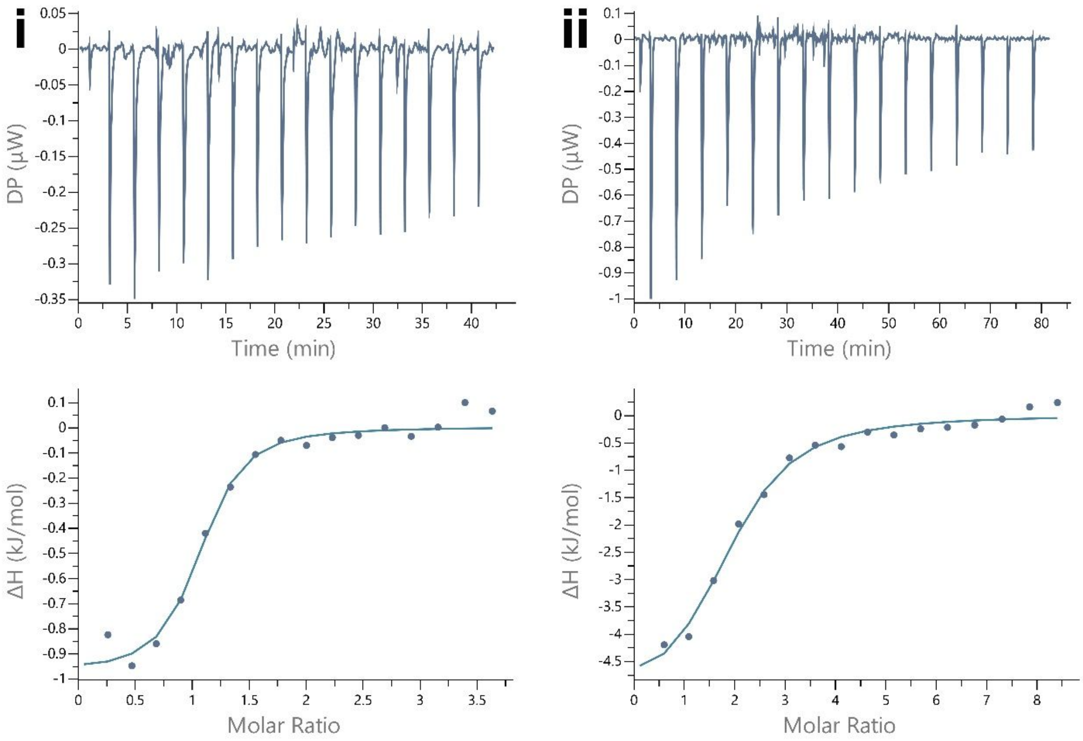

Figure S11A. ITC plots (heat flow *vs* time and molar enthalpy change *vs* molar ratio) of aptananozyme I, with L-DOPA (i) and D-DOPA (ii) in 5 mM MES pH 5.5, 5 mM $MgCl_2$, 100 mM NaCl.

The derived K_d values are summarized in Table S4.

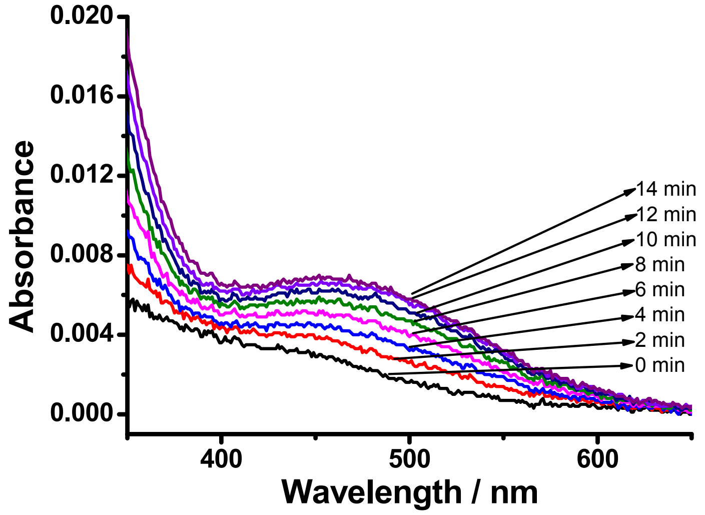

Figure S12. Time-dependent absorption spectra of oxidized L-tyrosinamide in the presence of 0.5 μg mL^{-1} aptananozyme X, 1 mM L-tyrosinamide, 5 mM H_2O_2, 5 mM AA^{-}.

.

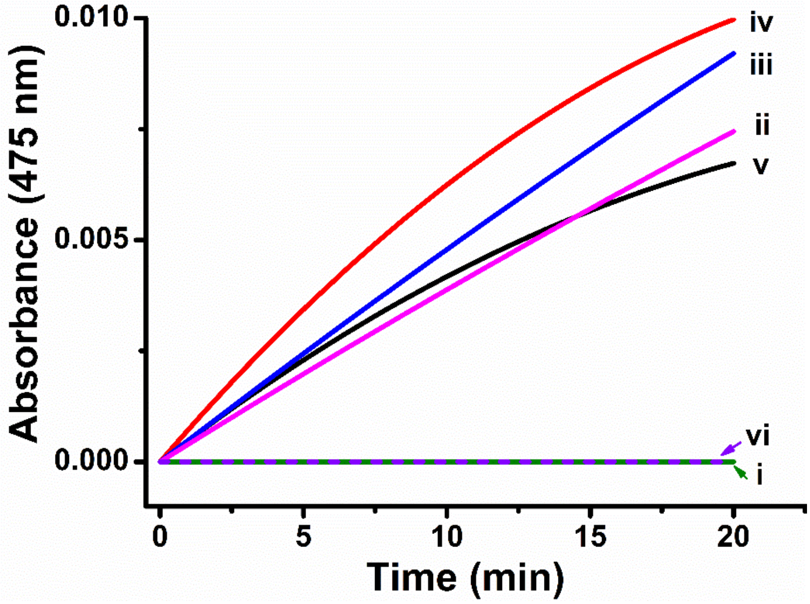

Figure S13. Time-dependent absorbance spectra upon oxidation of L-tyrosinamide to amidodopachrome in the presence of Cu^{2+}-ions-functionalized C-dots 0.5 μg mL^{-1}, 1 mM L-tyrosinamide, and 5 mM H_2O_2 using variable concentrations of ascorbate: (i) 0, (ii) 1, (iii) 2, (iv) 5, (v) 10 mM, the total volume of the reaction mixture: 100 μL. (vi) Time-dependent absorbance spectra upon oxidation of L-tyrosinamide to amidodopachrome in the presence of 0.5 μg mL^{-1} Cu^{2+}-modified C-dots, 1 mM L-tyrosinamide, 0 mM H_2O_2, 5 mM AA^{-}, the total volume of the reaction mixture: 100 μL.

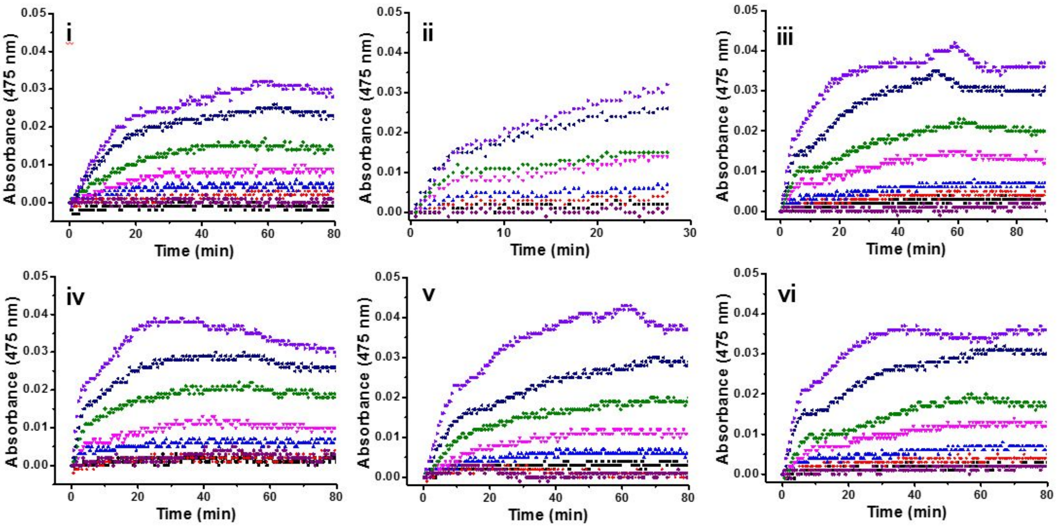

Figure S14. Time-dependent absorbance changes upon the oxidation of different concentrations of L-tyrosinamide to amidodopachrome by the aptananozymes X-XIV: (a) 0, (b) 25, (c) 50, (d) 100, (e) 250, (f) 500, (g) 1000, (h) 2000 μM. (i) Cu^{2+}-modified C-dots with scrambled TBA. (ii) aptananozyme X. (iii) aptananozyme XII. (iv) aptananozyme XIII. (v) aptananozyme XIV. (vi) aptananozyme XI. For all systems, the condition of the experiments corresponded to 0.5 μg mL^{-1} aptananozymes, 5 mM H_2O_2, and 5 mM AA^-.

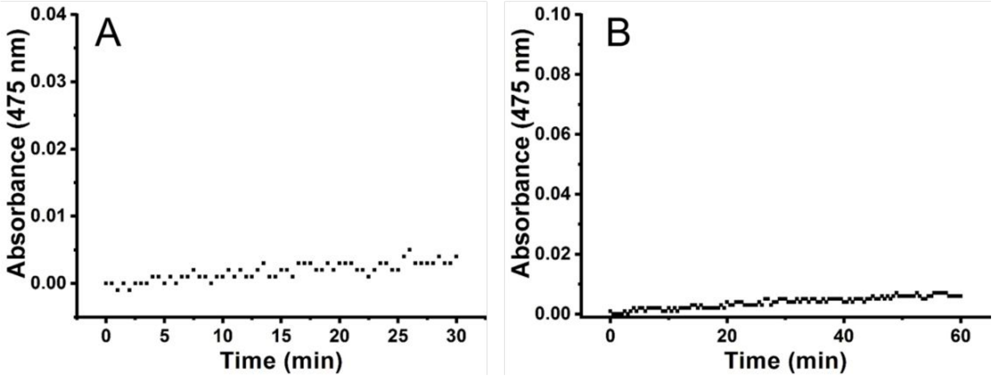

Figure S15 Time-dependent absorbance spectra upon oxidation of L-tyrosinamide in the presence of (A) C-dots 0.5 μg mL^{-1} and (B) Fe_3O_4 5 μg mL^{-1}, 1 mM L-tyrosinamide, 5 mM ascorbate, and 5 mM H_2O_2.

Figure S16 presents the raw heat plots (upper panels) and derived titration curves (lower panels) corresponding to (i) the scrambled TBA-aptananozyme and (ii)-(vi) the respective aptananozymes X-XIV.

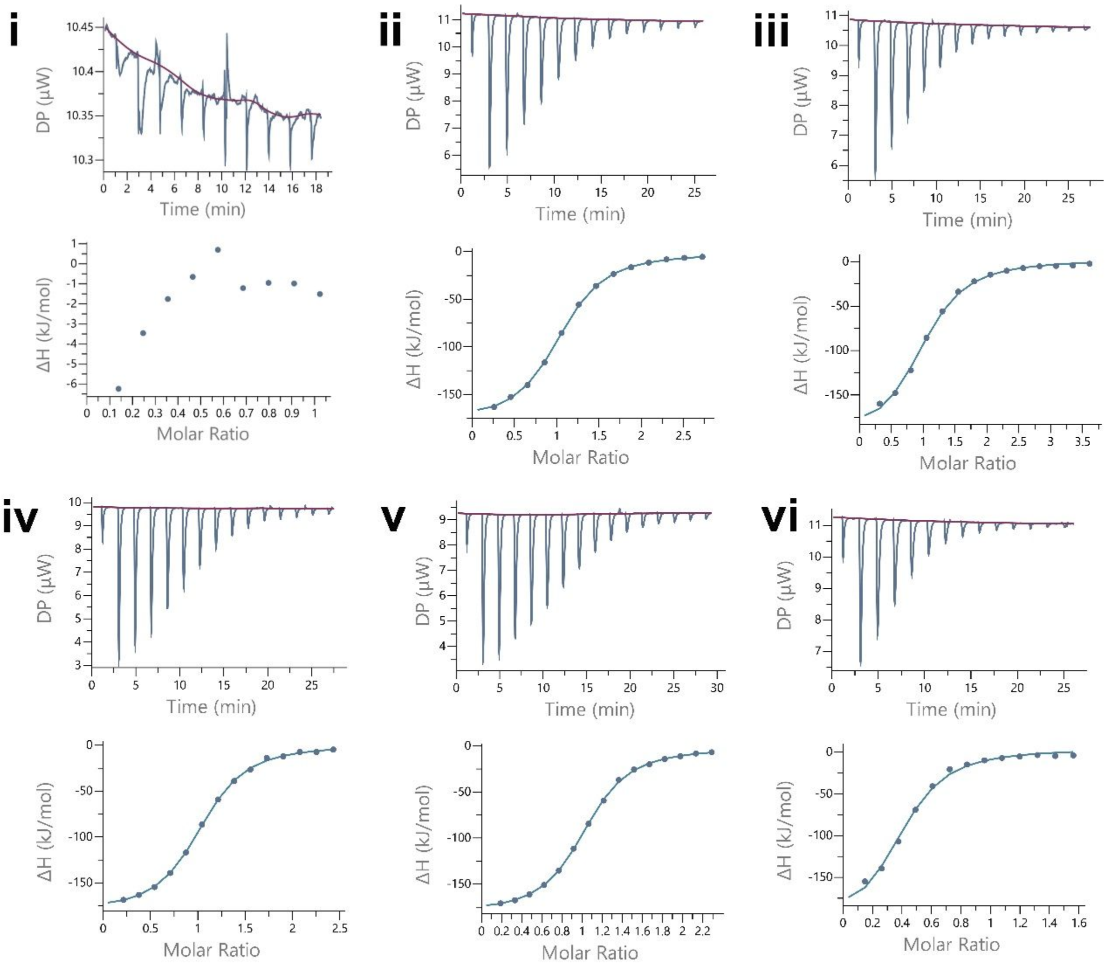

Figure S16. Isothermal titration calorimetry (ITC) titration plots (heat flow *vs* time and molar enthalpy change *vs* molar ratio) of different aptananozymes, with L-tyrosinamide in 50 mM PB buffer (pH 7.2, 50 mM NaCl, and 10 mM $MgCl_2$): (i) Cu^{2+}-modified C-dots with scrambled TBA. (ii) aptananozyme X. (iii) aptananozyme XI. (iv) aptananozyme XII. (v) aptananozyme XIII. (vi) aptananozyme XIV.

The derived K_d values are summarized in Table S6.

The chiral features of the tyrosinamide aptamer and its binding affinity towards L-tyrosine (K_d = 780 ± 11 µM) suggest that the modified Cu^{2+}-ions-functionalized C-dots, aptananozyme, should reveal enantioselective preference upon oxidation of a L-/D-racemic mixture in the presence of H_2O_2/ascorbate solution. Accordingly, we attempted to follow the enantioselective depletion of the racemic mixture of L-/D-tyrosine upon the aptananozyme-catalyzed oxidation of dopachrome in the presence of H_2O_2/ascorbate. While we encountered difficulties to follow the selective depletion of L-/D-tyrosine by liquid chromatography separation, due to limited solubility of L-/D-tyrosine in various solvents, and due to the presence of L-/D-tyrosine in the ascorbate-rich aqueous solution, we were able to follow the selective oxidation by applying a shift reagent (Europium tris[3-(heptafluoropropylhydroxymethylene)-(+)-camphorate]) to the reaction mixture Figure S17. The racemic L-/D-tyrosine mixture (1 mL, 1 mM each enantiomer) was subjected to the aptananozyme X (2 µg mL^{-1}) in a phosphate buffer solution that included ascorbate, 5 mM, and H_2O_2, 5 mM, for a time-interval of 3 hours. Subsequently, the aptananozyme particles were separated and the aqueous solution was evaporated to dryness, Dimethyl sulfoxide-d6, 0.75 ml that included the shift reagent, 2 mM, was added to the dried reaction product. The chemical shifts of the benzylic CH_2 protons were used to assess the selectivity of the substrate depletion upon oxidation. Control chemical shifts of the pure L-tyrosine, curve I, and pure D-tyrosine, curve II were used to identify the shift associated with the aptananozyme-driven depletion of the L-/D-tyrosine in the racemic mixture, curve III. One may realize the selective depletion of the L-tyrosine enantiomer in the racemic mixture occurred. By integration

of the respective bands, we estimate a *ca.* 80% excess of the L-tyrosine oxidation proceeded, demonstrating enantioselective oxidation of the L-tyrosine enantiomer in the racemic mixture.

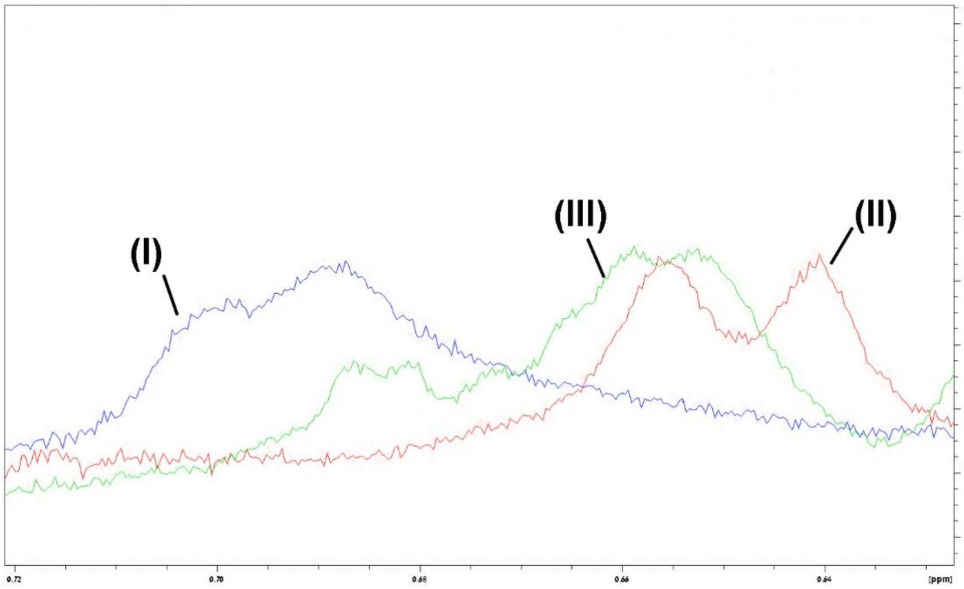

Figure S17. ^{1}H-NMR spectra (500 MHz) of pure L-tyrosine (I), pure D-tyrosine (II), and D-/L-tyrosine racemic mixture after catalytic reaction in d_6-DMSO.

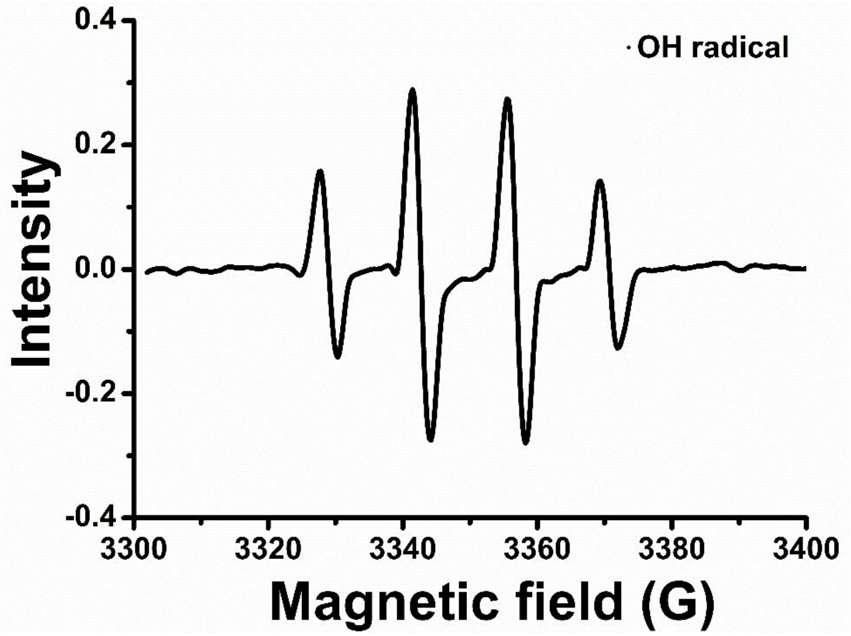

Figure S18. ESR spectrum corresponding to the •OH generated by the aptananozyme I in the presence of H_2O_2.

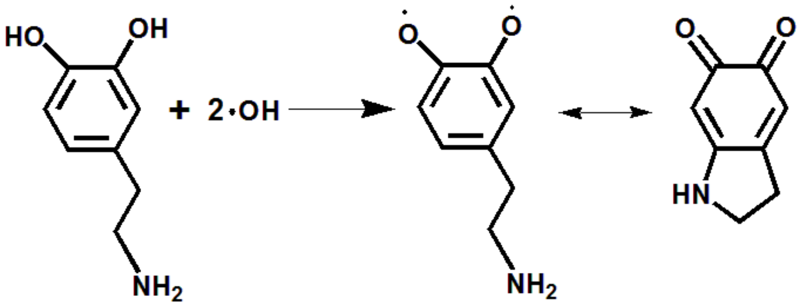

Figure S19. Oxidation process from dopamine to aminochrome in presence of dopamine aptananozyme and H_2O_2.

$$(1)\ Cu^{II} + AA^- \longrightarrow Cu^{I} + AA\cdot$$

$$(2)\ AA\cdot + H_2O_2 \longrightarrow AA^- + \cdot OOH + H^+$$

$$(3)\ Cu^{I} + H_2O_2 \longrightarrow Cu^{II} + \cdot OH + OH^-$$

$$(4)\ \cdot OH + AA^- \longrightarrow OH^- + AA\cdot$$

Figure S20. A set of reactions suggested as a possible route for the Cu^{2+}-ions-functionalied C-dots-catalyzed generation of •OOH and accompanied AA•, in the presence of the H_2O_2/AA^- mixture.

In this scheme, eq. (1) consistent with the experimental results demonstrating the formation of AA• by the catalyst, Figure 5A, panel II. The AA• induced formation of •OOH in the presence of H_2O_2, eq. (2), is supported by the formation of •OOH and AA•, Figure 5A, panel III. The resulting Cu^{I} species are oxidized by H_2O_2 to form the •OH that is rapidly depleted by the generation of AA•, eq. (3), and eq. (4), and the resulting AA• is feedback into reaction (2) that generates the peroxy •OOH oxygenation species.

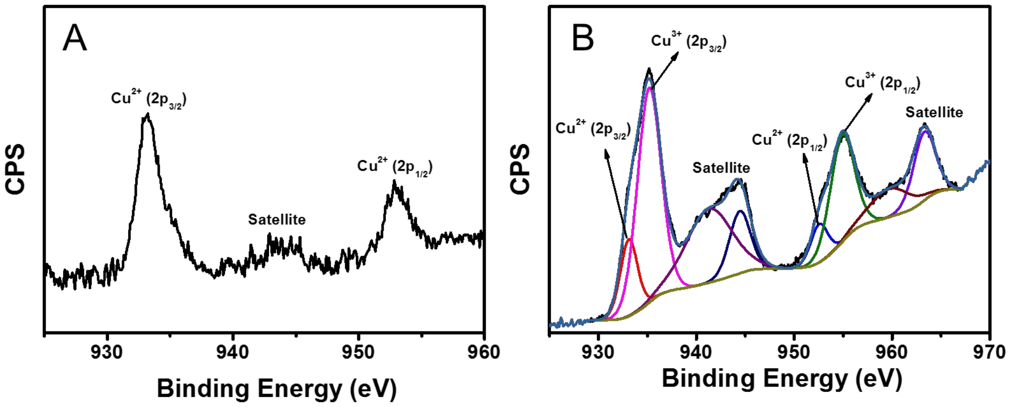

Figure S21. High-resolution XPS spectra of Cu (A, Cu^{2+}-ions-functionalized C-dots before reacting with AA^- and H_2O_2; B, Cu^{2+}-ions-functionalized C-dots after reacting with AA^- and H_2O_2).

Table S1. Concentration ratio of C-dots and DBA, and the loading of Cu^{2+}-ions on the C-dots, in different configuration of Aptananozymes.

Aptananozyme	*[C-dots]:[DBA]*	$m_{(C-dots)} : m_{Cu^{2+}}$ (mg)
IV	1:4.2 ± 0.2	1:0.04 ± 0.01
III	1:4.3 ± 0.3	1:0.04 ± 0.01
I	1:4.1 ± 0.2	1:0.04 ± 0.01
II	1:4.5 ± 0.1	1:0.04 ± 0.01
V	1:4.4 ± 0.1	1:0.04 ± 0.01
Scrambled	1:4.2 ± 0.3	1:0.04 ± 0.01

Table S2. Dissociation constants of the aptananozymes I-V and control system.

Aptananozyme	K_d [μM]	K_d [μM] (Aptamer only)
IV	1.02 ± 0.08	1.09 ± 0.06
III	1.06 ± 0.05	1.09 ± 0.04
I	0.98 ± 0.06	1.07 ± 0.04
II	3.6 ± 0.2	1.42 ± 0.05
V	3.9 ± 0.2	1.17 ± 0.07
Scrambled	N/A	N/A

Table S3. Kinetic parameters corresponding to the aptananozymes I by using substrates of L-DOPA and D-DOPA.

Substrate	Aptananozyme	V_{max} (μM min^{-1})	K_M (μM)	k_{cat} (10^{-4} s^{-1})	k_{cat}/K_M (s^{-1} M^{-1})
L-DOPA	I	0.12±0.01	139 ± 33	1.2 ± 0.1	0.86
D-DOPA	I	0.06±0.01	292 ± 45	0.6 ± 0.1	0.21

Table S4. Dissociation constants of the aptananozyme I toward L-DOPA and D-DOPA.

Substrate	Aptananozyme	K_d [μM]
L-DOPA	I	1.7 ± 0.4
D-DOPA	I	6.6 ± 1.0

Table S5. Concentration ratio of C-dots and TBA, and the loading of Cu^{2+}-ions on the C-dots, in different configurations of Aptananozymes.

Aptananozyme	*[C-dots]:[TBA]*	$m_{(C-dots)} : m_{Cu^{2+}}\ (mg)$
IV	1:4.3 ± 0.1	1:0.04 ± 0.01
III	1:4.1 ± 0.2	1:0.04 ± 0.01
I	1:4.5 ± 0.2	1:0.04 ± 0.01
II	1:4.3 ± 0.1	1:0.04 ± 0.01
V	1:4.1 ± 0.3	1:0.04 ± 0.01
Scrambled	1:4.1 ± 0.25	1:0.04 ± 0.01

Table S6. Dissociation constants of the aptananozymes (X)-(XIV) and control system.

Aptananozyme	K_d [μM]
XIII	0.77 ± 0.03
XII	0.81 ± 0.04
X	0.83 ± 0.01
XI	1.24 ± 0.05
XIV	1.4 ± 0.1
Scrambled	N/A

Reference.

1. Qu, S.; Wang, X.; Lu, Q.; Liu, X.; Wang, L. A Biocompatible Fluorescent Ink Based on Water-Soluble Luminescent Carbon Nanodots. *Angew. Chem., Int. Ed.* **2012**, *124*, 12381-12384.
